# Evaluation of cultivated and wild genotypes of *Lens* species under alkalinity stress and their molecular collocation using microsatellite markers

**DOI:** 10.1371/journal.pone.0199933

**Published:** 2018-08-13

**Authors:** Dharmendra Singh, Chandan Kumar Singh, Yash Pal Singh, Vijayata Singh, Rajendra Singh, Ram Sewak Singh Tomar, Satish Kumar Sanwal, Sourabh Karwa, Vinay Kumar Mishra, Susheel Kumar Sarkar, Madan Pal, Arun Kumar, Rajendra Kumar Yadav, Parbodh Chander Sharma

**Affiliations:** 1 Division of Genetics, ICAR-Indian Agricultural Research Institute, New Delhi, India; 2 ICAR-Regional Station of Central Soil Salinity Research Institute, Lucknow, India; 3 ICAR- Central Soil Salinity Research Institute, Karnal, India; 4 Divisions of Soil Science and Agricultural Chemistry, ICAR-Indian Agricultural Research Institute, New Delhi, India; 5 ICAR-National Research Centre on Plant Biotechnology, Pusa Campus, New Delhi, India; 6 Division of Plant Physiology, ICAR-Indian Agricultural Research Institute, New Delhi, India; 7 ICAR-Indian Agricultural Statistical Research Institute, New Delhi, India; 8 National Phytotron Facility, ICAR-Indian Agricultural Research Institute, New Delhi, India; 9 Department of Genetics and Plant Breeding, Chandra Shekhar Azad University of Agriculture and Technology, Kanpur, India; Jawaharlal Nehru University, INDIA

## Abstract

In this study, 285 lentil genotypes were phenotyped under hydroponic and alkaline field conditions. Significant genotypic variation for alkalinity stress was observed among the six *Lens* species screened hydroponically and in the field having pH up to 9.1. The crucial parameters, like whole Na^+^ and K^+^ contents and the Na^+^/K^+^ ratio at 40 mM NaHCO_3_ were found significantly correlated with seedling survivability under hydroponics (r = -0.95, r = 0.93 and -0.97). Genotypes, ranked on the bases of seed yield, restricted uptake of Na^+^ with thick pith area, increased vascular bundles, less H_2_O_2_ production and low Na^+^/K^+^ ratio, were found important physio-anatomical traits for alkalinity stress tolerance. The proper regulation of Na^+^ uptake was found for maintaining higher K^+^. This relationship is probably the main factor responsible for a better mechanism for tolerance to high pH up to 9.1 in tolerant breeding lines PDL-1 and PSL-9 (cultivars) and ILWL-15, ILWL-192 and ILWL-20 (wild accessions). Based on UPGMA dendrogram, all the genotypes were clustered into four diverse groups. DMRT was implied within the group to differentiate genotypes based on phenotypic response under alkalinity stress. These results can be utilized for selecting diverse parents for developing alkalinity tolerant genotypes.

## Introduction

Soil salinity and alkalinity are common severe constraints to crop productivity. In the world, about 830 million hectares of the area are affected by salinity and alkalinity, out of which 434 million hectares area is alkaline [1]. Alkaline soil is generally dominated by excess sodium on exchange sites and has a high concentration of carbonate/bicarbonate anions, which adversely affect the physical and nutritional properties of the soil. The carbonate/bicarbonate ions are major contributors of soil alkalinity. Presence of these in excess Na^+^, high pH and osmotic stress lead to drastic reduction in plant growth and development. One of the major approaches used to manage alkaline soils and water, is to develop alkalinity tolerant genotypes. This can be achieved by harnessing inter-specific or intra-specific variability.

Lentil (*Lens culinaris* Medikus), is an important legume, providing quality protein, carbohydrates, fibre and minerals for the humans and fodder for livestock. Globally, it is cultivated on 3.6 million ha area with a production of 3.4 MT [2]. However, it is an alkaline sensitive crop and yield is drastically reduced under high alkalinity. High levels of alkalinity stress in soil or irrigation water adversely affect seed germination, growth and productivity [3, 4]. The most evident symptoms of alkaline stress on plants are the induction of leaf chlorosis and stunted growth due to higher uptake of Na^+^ and lower uptake of nutrients [5, 6, 7]. Salinity/alkalinity also distorts anatomical structures. A major reduction in the dimension of vascular tissue was also observed in the roots of *Chloris gayana* Kunth under salinity stress [8]. Limited information is available on the anatomical deformity of legumes like pea [9], kidney bean [10] and lotus [11] due to alkalinity stress.

Screening of germplasm at seedling stage under the hydroponic condition is a readily acceptable tool as it is based on a simple criterion of selection [12]. Screening for alkalinity tolerance should also be carried out in the field at sites where alkalinity stress is a problem. However, screening in the field may be difficult because of heterogeneity and is also influenced by a large number of environmental factors. These difficulties can be overcome by using hydroponic based screening system as it is more effective as it provides consistent control over pH in crop plants [12, 13]. Hydroponic screening system has been found most convenient and scientific because seedlings require less space and tolerant ones may be recovered for seed production purpose. There can also be possibility of pre-selection of breeding lines, progenies and cultivars before field evaluation. Evaluation of crop plants for tolerance to alkalinity stress has been well documented in mustard [12] and bean lines [14] at early growth stage under hydroponic and alkaline field conditions, respectively. However, initial screening in hydroponic and later in field conditions can be the best strategy for accurate phenotyping of alkalinity tolerance. There are various reports published on the effects of salinity stress on plants, but limited information is available on the effects of alkalinity stress.

Molecular analysis of characterized genotypes can be directly utilized by lentil breeders to select parents for contrasting characters associated with alkalinity stress tolerance. Different types of molecular markers have been successfully used for identification of genotypes, diversity and gene/QTL analysis in lentil [15, 16, 17, 18]. Simple sequence repeat (SSR) markers have been extensively used for the assessment of genetic diversity in lentil [19, 20, 21] as they are efficient, easy to use and have high reproducibility and co-dominance. However, no work has been done so far on the evaluation of *Lens* species by using morpho-anatomical and physiological traits and molecular markers. Therefore, this study was planned with following objectives: (1) morho-anatomical and physiological characterization of genotypes for alkalinity stress tolerance under hydroponics, (2) field validation of genotypes for seed yield and physiological characterization for alkalinity tolerance and (3) diversity analysis of genotypes using microsatellite markers.

## Material and methods

### Plant materials

The experiment was conducted at National Phytotron Facility, ICAR-Indian Agricultural Research Institute, New Delhi, India. Two hundred eighty five genotypes were used for selection of diverse genotypes under alkalinity stress conditions. The details such as origin of country, reaction to the alkalinity of genotypes are presented in Table 1. The wild accessions included were *L*. *orientalis*, *L*. *odomensis*, *L*. *nigricans*, *L*. *ervoides* and *L*. *lamottei*, the first two belong to the primary gene pool, while the last three to the secondary/tertiary gene pool. Air temperature in the National Phytotron Facility was 22/18°C (±2°C), day/night; photoperiod was 10/14 h (light/dark) and the relative humidity was approximately 45%.

**Table 1:** Genotypes with different origins and sensitivity to alkalinity stress.

S. No.	Genotype	Origin	Type	Alk R	S. No.	Genotype	Origin	Type	Alk R
1	121–12	India	GC	S	144	ILL-590	Turkey	GC	S
2	1220–11	India	BL	MT	145	ILL-6002	ICARDA	GC	MT
3	210–11	India	BL	S	146	ILL-7349	Nepal	GC	S
4	330–12	India	GC	S	147	ILL-76037	ICARDA	GC	S
5	BM-4	Bangladesh	Cult.	S	148	ILL-7978	ICARDA	GC	S
6	DPL-62	India	Cult.	S	149	ILL-7979	ICARDA	GC	S
7	E-153	India	GC	S	150	ILL-7982	ICARDA	GC	S
8	FLIP-96-51	ICARDA	GC	S	151	ILL-8006	Bangladesh	GC	S
9	IG-109039	ICARDA	GC	S	152	ILL-8108	Argentina	GC	S
10	IG-111991	ICARDA	LR	S	153	ILL-8329	ICARDA	GC	S
11	IG-111996	ICARDA	LR	S	154	ILL-91887	ICARDA	GC	S
12	IG-112078	ICARDA	LR	S	155	ILL-9841	ICARDA	GC	S
13	IG-11210	ICARDA	LR	S	156	ILL-9900	ICARDA	GC	S
14	IG-112128	ICARDA	LR	S	157	ILL-9916	ICARDA	GC	S
15	IG-112131	ICARDA	LR	S	158	ILL-9941	ICARDA	GC	S
16	IG-112137	ICARDA	LR	S	159	ILL-9960	ICARDA	GC	S
17	IG-116551	ICARDA	LR	S	160	ILWL-06	Turkey	Wild	S
18	IG-129185	ICARDA	LR	S	161	ILWL-09	Syria	Wild	S
19	IG-129214	ICARDA	LR	S	162	ILWL-10	-	Wild	S
20	IG-129287	ICARDA	LR	S	163	ILWL-100	Turkey	Wild	S
21	IG-129291	ICARDA	LR	S	164	ILWL-104	Turkey	Wild	S
22	IG-129293	ICARDA	LR	S	165	ILWL-125	Syria	Wild	S
23	IG-129302	ICARDA	LR	S	166	ILWL-128	Syria	Wild	S
24	IG-129309	ICARDA	LR	S	167	ILWL-13	Italy	Wild	S
25	IG-129313	ICARDA	LR	S	168	ILWL-133	Syria	Wild	S
26	IG-129315	ICARDA	LR	S	169	ILWL-137	Syria	Wild	S
27	IG-129317	ICARDA	LR	S	170	ILWL-142	Syria	Wild	S
28	IG-129319	ICARDA	LR	S	171	ILWL-15	France	Wild	T
29	IG-129372	ICARDA	LR	S	172	ILWL-165	Syria	Wild	S
30	IG-129560	ICARDA	LR	S	173	ILWL-184	Syria	Wild	S
31	IG-12970	ICARDA	LR	S	174	ILWL-185	Syria	Wild	S
32	IG-130033	ICARDA	LR	S	175	ILWL-192	Syria	Wild	T
33	IG-130219	ICARDA	LR	S	176	ILWL-20	Palestine	Wild	T
34	IG-130272	ICARDA	LR	S	177	ILWL-203	Turkey	Wild	S
35	IG-134342	ICARDA	LR	S	178	ILWL-221	Turkey	Wild	S
36	IG-134347	ICARDA	LR	S	179	ILWL-227	Syria	Wild	S
37	IG-134356	ICARDA	LR	S	180	ILWL-23	Italy	Wild	S
38	IG-135424	-	Wild	S	181	ILWL-237	Syria	Wild	S
39	IG-135428	-	Wild	S	182	ILWL-238	Syria	Wild	S
40	IG-136607	ICARDA	LR	S	183	ILWL-253	Syria	Wild	S
41	IG-136608	-	Wild	S	184	ILWL-269	Turkey	Wild	S
42	IG-136612	Turkey	Wild	S	185	ILWL-29	Spain	Wild	S
43	IG-136614	Italy	Wild	S	186	ILWL-292	Turkey	Wild	MT
44	IG-136618	Croatia	Wild	S	187	ILWL-3	Turkey	Wild	S
45	IG-136620	Slovenia	Wild	S	188	ILWL-314	Turkey	Wild	S
46	IG-136626	Israel	Wild	S	189	ILWL-320	Turkey	Wild	MT
47	IG-136637	France	Wild	S	190	ILWL-321	Turkey	Wild	MT
48	IG-136652	Israel	Wild	S	191	ILWL-334	Jordan	Wild	S
49	IG-136653	Israel	Wild	S	192	ILWL-377	Tajiskistan	Wild	S
50	IG-136673	Turkey	Wild	S	193	ILWL-340	Jordan	Wild	S
51	IG-136788	Syria	Wild	S	194	ILWL-35	Turkey	Wild	S
52	IG-140910	Azerbaijan	Wild	S	195	ILWL-350	Syria	Wild	S
53	IG-149	ICARDA	LR	S	196	ILWL-357	Syria	Wild	S
54	IG-129304	ICARDA	LR	S	197	ILWL-361	Syria	Wild	S
55	IG-49	ICARDA	LR	S	198	ILWL-362	Syria	Wild	S
56	IG-5320	ICARDA	LR	S	199	ILWL-366	Syria	Wild	S
57	IG-69540	ICARDA	LR	S	200	ILWL-370	Syria	Wild	S
58	IG-69549	ICARDA	LR	S	201	ILWL-398(A)	Lebanon	Wild	S
59	IG-70174	ICARDA	LR	S	202	ILWL-401	Lebanon	Wild	S
60	IG-70230	ICARDA	LR	S	203	ILWL-415	Syria	Wild	MT
61	IG-71352	ICARDA	LR	S	204	ILWL-418	Syria	Wild	S
62	IG-71630	ICARDA	LR	S	205	ILWL-428	Spain	Wild	S
63	IG-71646	ICARDA	LR	S	206	ILWL-430	Spain	Wild	S
64	IG-71685	ICARDA	LR	S	207	ILWL-436	Turkey	Wild	MT
65	IG-71710	ICARDA	LR	S	208	ILWL-437	Turkey	Wild	S
66	IG-73717	ICARDA	LR	S	209	ILWL-438	Turkey	Wild	S
67	IG-73798	ICARDA	LR	S	210	ILWL-44	Slovenia	Wild	S
68	IG-73802	ICARDA	LR	S	211	ILWL-447	Turkey	Wild	S
69	IG-73816	ICARDA	LR	S	212	ILWL-462	Turkey	Wild	S
70	IG-73945	ICARDA	LR	S	213	ILWL-464	Syria	Wild	S
71	IG-75920	ICARDA	LR	S	214	ILWL-472	-	Wild	S
72	IG-9	ICARDA	LR	S	215	ILWL-55(2)	Israel	Wild	S
73	IG-936	ICARDA	LR	S	216	ILWL-58	Turkey	Wild	S
74	ILL-10030	ICARDA	GC	S	217	ILWL-60	Turkey	Wild	S
75	ILL-10031	ICARDA	GC	S	218	ILWL-69	Uzbekistan	Wild	S
76	ILL-10032	ICARDA	GC	S	219	ILWL-83	Turkey	Wild	S
77	ILL-10034	ICARDA	GC	S	220	ILWL-95	Turkey	Wild	S
78	ILL-10040	ICARDA	GC	S	221	IPL-406	India	Cult.	S
79	ILL-10041	ICARDA	GC	S	222	JL-3	India	Cult.	S
80	ILL-10043	ICARDA	GC	S	223	L-404	India	BL	S
81	ILL-10056	ICARDA	GC	S	224	L-4076	India	Cult.	S
82	ILL-10061	ICARDA	GC	S	225	L-4078	India	BL	S
83	ILL-10062	ICARDA	GC	S	226	L-4147	India	Cult.	S
84	ILL-10063	ICARDA	GC	S	227	L-4578	India	BL	S
85	ILL-10074	ICARDA	GC	S	228	L-4590	India	Cult.	S
86	ILL-10075	ICARDA	GC	S	229	L-4594	India	Cult.	S
87	ILL-10082	ICARDA	GC	S	230	L-4602	India	BL	S
88	ILL-10133	ICARDA	GC	S	231	L-4603	India	BL	S
89	ILL-10234	ICARDA	GC	S	232	L-4605	India	BL	S
90	ILL-10266	ICARDA	GC	S	233	L-4618	India	BL	MT
91	ILL-10270	ICARDA	GC	S	234	L-4619	India	BL	S
92	ILL-1046	ICARDA	GC	S	235	L-4620	India	BL	S
93	ILL-10756	ICARDA	GC	S	236	L-4650	India	BL	S
94	ILL-10794	ICARDA	GC	S	237	L-4701	India	BL	S
95	ILL-10795	ICARDA	GC	S	238	L-5253	India	BL	S
96	ILL-10804	ICARDA	GC	S	239	L-7752	India	BL	S
97	ILL-10805	ICARDA	GC	S	240	L-7818	India	BL	S
98	ILL-10806	ICARDA	GC	S	241	L-7903	India	BL	MT
99	ILL-10807	ICARDA	GC	S	242	L-7905	India	BL	S
100	ILL-10809	ICARDA	GC	S	243	L-7920	India	BL	S
101	ILL-10810	ICARDA	GC	S	244	LC-270-804	India	BL	S
102	ILL-10811	ICARDA	GC	S	245	LC-282-1077	India	BL	S
103	ILL-10812	ICARDA	GC	S	246	LC-282-1110	India	BL	MT
104	ILL-10817	ICARDA	GC	S	247	LC-282-1444	India	BL	S
105	ILL-10818	ICARDA	GC	S	248	LC-282-896	India	BL	S
106	ILL-10819	ICARDA	GC	S	249	LC-284-116	India	BL	S
107	ILL-10820	ICARDA	GC	S	250	LC-284-1209	India	BL	S
108	ILL-10823	ICARDA	GC	S	251	LC-285-1344	India	BL	S
109	ILL-10826	ICARDA	GC	S	252	LC-289-1444	India	BL	S
110	ILL-10827	ICARDA	GC	S	253	LC-289-1447	India	BL	S
111	ILL-10831	ICARDA	GC	S	254	LC-292-1485	India	BL	S
112	ILL-10834	ICARDA	GC	S	255	LC-292-1544	India	BL	S
113	ILL-10835	ICARDA	GC	S	256	LC-292-997	India	BL	S
114	ILL-10836	ICARDA	GC	S	257	LC-300-1	India	BL	S
115	ILL-10837	Turkey	GC	S	258	LC-300-11	India	BL	S
116	ILL-10848	Bangladesh	GC	S	259	LC-300-12	India	BL	S
117	ILL-10857	ICARDA	GC	S	260	LC-300-13	India	BL	S
118	ILL-10893	ICARDA	GC	S	261	LC-300-15	India	BL	MT
119	ILL-10894	ICARDA	GC	S	262	LC-300-16	India	BL	S
120	ILL-10897	ICARDA	GC	S	263	LC-300-2	India	BL	S
121	ILL-10913	ICARDA	GC	S	264	LC-300-3	India	BL	S
122	ILL-10915	ICARDA	GC	S	265	LC-300-4	India	BL	S
123	ILL-10917	ICARDA	GC	S	266	LC-300-6	India	BL	S
124	ILL-10921	ICARDA	GC	S	267	LC-300-7	India	BL	MT
125	ILL-10922	ICARDA	GC	S	268	LC-300-8	India	BL	S
126	ILL-10951	ICARDA	GC	S	269	LC-300-9	India	BL	MT
127	ILL-10953	ICARDA	GC	S	270	LC-74-1-51	India	BL	S
128	ILL-10960	ICARDA	GC	S	271	PAL-3	ICARDA	GC	MT
129	ILL-10961	ICARDA	GC	S	272	PDL-1	ICARDA	BL	T
130	ILL-10963	ICARDA	GC	S	273	PDL-2	ICARDA	BL	MT
131	ILL-10964	ICARDA	GC	S	274	PKVL-1	India	Cult.	S
132	ILL-10965	ICARDA	GC	S	275	PL-1	India	Cult.	S
133	ILL-10967	ICARDA	GC	S	276	PL-4	India	Cult.	S
134	ILL-10969	ICARDA	GC	S	277	PL-406	India	Cult.	S
135	ILL-10970	ICARDA	GC	S	278	PL-5	India	Cult.	S
136	ILL-10972	ICARDA	GC	S	279	PSL-1	ICARDA	GC	MT
137	ILL-1970	Ethiopia	GC	S	280	PSL-7	ICARDA	GC	MT
138	ILL-358	Mexico	GC	S	281	PSL-9	India	BL	T
139	ILL-3829	ICARDA	GC	S	282	SEHORE-74-3	India	Cult.	S
140	ILL-4605	Argentina	Cult.	S	283	SKL-259	India	BL	S
141	ILL-560	Turkey	GC	MT	284	VL-507	India	Cult.	S
142	ILL-5722	ICARDA	GC	MT	285	WBL-77	India	Cult.	S
143	ILL-5883	Jordan	GC	S					

Alk R = Alkanity Reaction; GC = Germplasm collection; BL = Breeding Lines; LR = Land races; S = Sensitive; T = Tolerant; MT = Moderately Tolerant in Alk R

### Evaluation of genotypes under alkalinity stress

Seeds of all the genotypes were sterilized in sodium hypochlorite (1%) for 2-3min then washed three times with distilled water. Twenty seeds of each genotype were exposed to 20 and 40 mM concentration of sodium carbonate (NaHCO_3_) along with a control for evaluation for germination. The germination was recorded at 12 h interval for 10 days. Seeds were considered germinated, when the radicle had emerged and attained 1 mm length. Germination percentage was calculated with the formula: Germination percent = (Total number of seeds germinated / Total number of seeds sown) x 100.

The *Lens* species were also assessed for alkalinity stress tolerance at seedling stage. Seven days old seedlings of cultivated and 14 d old seedlings of wild species were transferred to hydroponic medium and buffered with 20 mM and 40 mM sodium bicarbonate (NaHCO_3_) to obtain different pH levels. The pH of the nutrient solution was obtained to 8.8 and 9.1 and maintained constant using 1M KOH on a daily basis. The nutrient solution without bicarbonate was used as control (pH 7.2) which was prepared as per nutrient composition suggested by Javid et al. [12]. The solution was renewed at 2d interval to maintain nutrient concentration. The solution was regularly aerated by bubbling air with an aquarium pump over 15 d. After 15 d of alkalinity stress (20 and 40 mM NaHCO_3_), data were recorded on visual salt injuries, seedling survivability, seedling growth (root and shoot length) and biomass accumulation (fresh and dry weight of roots and shoots). All the genotypes were grouped into three categories according to their response to alkalinity stress on plant survival at 20 and 40 mM NaHCO_3_ concentrations as per the following criteria: (1) tolerant genotypes, showing 100% plant survival at 20 mM and ≤61% plant survival at 40 mM NaHCO_3,_ (2) moderately tolerant genotypes, showing ≤87% survival at 20 mM, whereas in case of 40 mM the survival was only ≤50%, (3) sensitive genotypes showing ≤67% plant survival at 20 mM and no plant survival at 40 mM NaHCO_3_. The survived plants were kept in the hydroponics without alkalinity stress for a week and then transferred to field at normal conditions till the maturity for evaluation in terms of seed yield/plant. For comparison, the control plants (without stress) were also transferred to the field for recording seed yield per plant. The reduction percent in yield was calculated by the formula: (seed yield of stressed plants-seed yield of control plants)/ (seed yield of control plants) x 100. Alkalinity stress tolerance was also estimated on the basis of visual salt injury of plants using 1–5 score scale, where: 1 = healthy plants with no visible symptoms of alkalinity stress, 2 = green plants with slight wilting, 3 = leaves turning yellowish green with moderate wilting, 4 = leaves yellowish brown with severe wilting and 5 = partial and completely dried leaves and/or stem. The scores were averaged and used for the assessment of alkalinity stress tolerance level of genotypes. Each treatment was replicated three times, with six plants in each treatment. At each sampling, plants were separated into roots and shoots. To remove the external treatment solutions, roots were washed three times in distilled water. Surface water was blotted off using paper towels and fresh mass was measured. Tissues were oven dried at 65°C for 72 h and dry mass was determined.

#### Analysis of mineral

For mineral analysis, dry plant material of selected plants of control and treated genotypes were ground in a pestle and mortar and digested in H_2_SO_4_. Concentration of Na^+^ and K^+^ of the digest was determined by Flame Photometer (Systronics 128, India). Na^+^ and K^+^ were calculated on percent dry weight basis.

#### Detection of H_2_O_2_ level

A set of 15 alkaline treated root tips from each treatment were collected and washed in 200 mM CaCl_2_ solution for 10 min. These root tips were excised and placed into a solution containing 200 mM CaCl_2_ (pH 4.4) and 10 mM FDA (Fluorescein diacetate) for 15 min. The FDA fluorescence was then detected under a fluorescence microscope [22].

#### Root and shoot anatomy

For studying root anatomy under control and alkalinity stress, method followed by Krishnamurthy et al. [23] with slight modification was used. Free hand sections were cut and the selected sections were stained with 50% toluidine blue. These sections were washed to remove excess stain and then mounted in distilled water. Pictures were taken using an optical microscope (Zeiss, AXIOSKOP 2) at 10 × magnification.

### Evaluation of genotypes under alkaline field conditions

Field experiments were conducted at Central Soil Salinity Research Institute, Regional Research Station, Farm, Lucknow, India (80^0^ 46’ 32” E 26^o^ 47’ 45” N and 120 m above mean sea level and Chandra Shekhar Azad University of Agriculture and Technology, Research Farm, Kanpur, India (26.4912° N, 80.3073° E and 125.9 on above mean sea level) during 2013–14 and 2014–15. The initial soil pH during 2013–14 at Lucknow and Kanpur sites was 9.0 and 9.1 with the exchangeable sodium percentage (ESP) 22.0 and 24.5, respectively. During 2014–2015, the pH value at the experimental farm at Lucknow was 9.5 with the corresponding ESP 40.0. The pH of normal soil was 7.6 and ESP 10.5 at Kanpur. To record seed yield per plant, a total of 236 and 224 genotypes were tested at Kanpur (pH 9.1) during the year 2013–14 and 2014–15, respectively. Similarly, 224 genotypes were evaluated during 2013–14 and 2014–15 at Lucknow under pH of 9.0 and 9.5, respectively. The variation in a number of genotypes was due to an insufficient number of seeds produced by wild accessions for further evaluation under alkaline stress. Ten plants from each genotype were harvested at 75 percent physiological maturity to record the seed yield. The total rainfall during cropping season of 2013–14 and 2014–15 at Kanpur and Lucknow was 148.8 mm and 212.0 mm and 90.2 mm and 72.6 mm, respectively. The maximum average temperature during 2013–14 and 2014–15 at Kanpur and Lucknow was 23.9°C and 21.4°C and 24.7°C and 24.2°C and the minimum 8.7°C and 14.4°C and 11.8°C and 10.8°C, respectively. The crop was raised with recommended cultural practices. Plants were sampled for Na^+^ and K^+^ analyses at seedling and flowering stages and seed yield was recorded at maturity. Na^+^ and K^+^ were estimated following the method of Jackson’s using a Flame photometer [24].

### Molecular analysis

DNA was extracted using modified CTAB method [25] and was quantified using a spectrophotometer. Thirty SSR markers which were polymorphic between two genotypes that a showed contrasting response to alkalinity stress, along with additional 38 arbitrary SSR markers were used for screening 285 genotypes. These markers were selected based on earlier lentil reports published [16, 26, 27].

PCR amplifications were performed in 10μl reaction volume, consisting of 1 X PCR buffer, 1.5 mM MgCl_2_ and 0.5 μM primers each of forward and reverse, 1 mM dNTP, 0.5 U *Taq* DNA polymerase and 50 ng template DNA. PCR cycling conditions were as follows: Pre-denaturation at 94°C for 3 min followed by 40 cycles of denaturation at 94°C for 30 sec, annealing at 55°C for 30 sec, elongation at 72°C for 1 min with a final extension at 72°C for 10 min. PCR amplified products were separated on 3% ultra high resolution agarose gels and documented using Vilber Lourmat Gel Documentation System.

#### Genetic diversity analysis

The genetic profile of 285 genotypes was scored on the basis of difference in allele size using 30 SSR markers which were polymorphic to alkalinity tolerant and sensitive genotype and then compared with the results obtained by adding 38 more SSR markers which were selected arbitrarily. The major allele frequency, PIC and genetic distance based clustering were performed with Unweighted Pair Group Method for Arithmetic average (UPGMA) using Power Marker v3.25 software [28] and the dendrogram was constructed following bootstrap analysis with 1000 permutations for all the genotypes using MEGA 4.0 software [29]. The population structure was determined on 285 genotypes comprising both wild and cultivated ones using Structure 2.3.4 software [30]. The number of subgroup K was estimated by 7 independent runs for each K (1 to 15) using the admixed model with 1,00,000 Monte Carlo Markov Chain (MCMC) replicates after a burn-in of 10,000 replicates. The structure outputs were visualized using Structure Harvester from which Evano plots were constructed [31].

#### Statistical analysis

Duncan’s Multiple Range Test (DMRT) (*P* = 0.05) was followed to find out differences among clusters for significance by using SAS 9.4 software. Data for morpho-physiological traits were analysed using two-way ANOVA to determine if significant differences were present among the means. Variances were checked by plotting residual vs. fitted values to confirm the homogeneity of the data. Differences among the mean values were assessed by Least Significant Differences (LSD). Relationships between individual variables were examined using simple correlations which were also performed using SAS 9.4 software. Spearman’s rank correlation test (rs) was used to examine consistency in the rankings of genotypes for alkalinity tolerance and seed yield between the hydroponic and field experiments.

## Results

### Phenotyping of alkalinity stress tolerance under hydroponics

**Fig 1:**
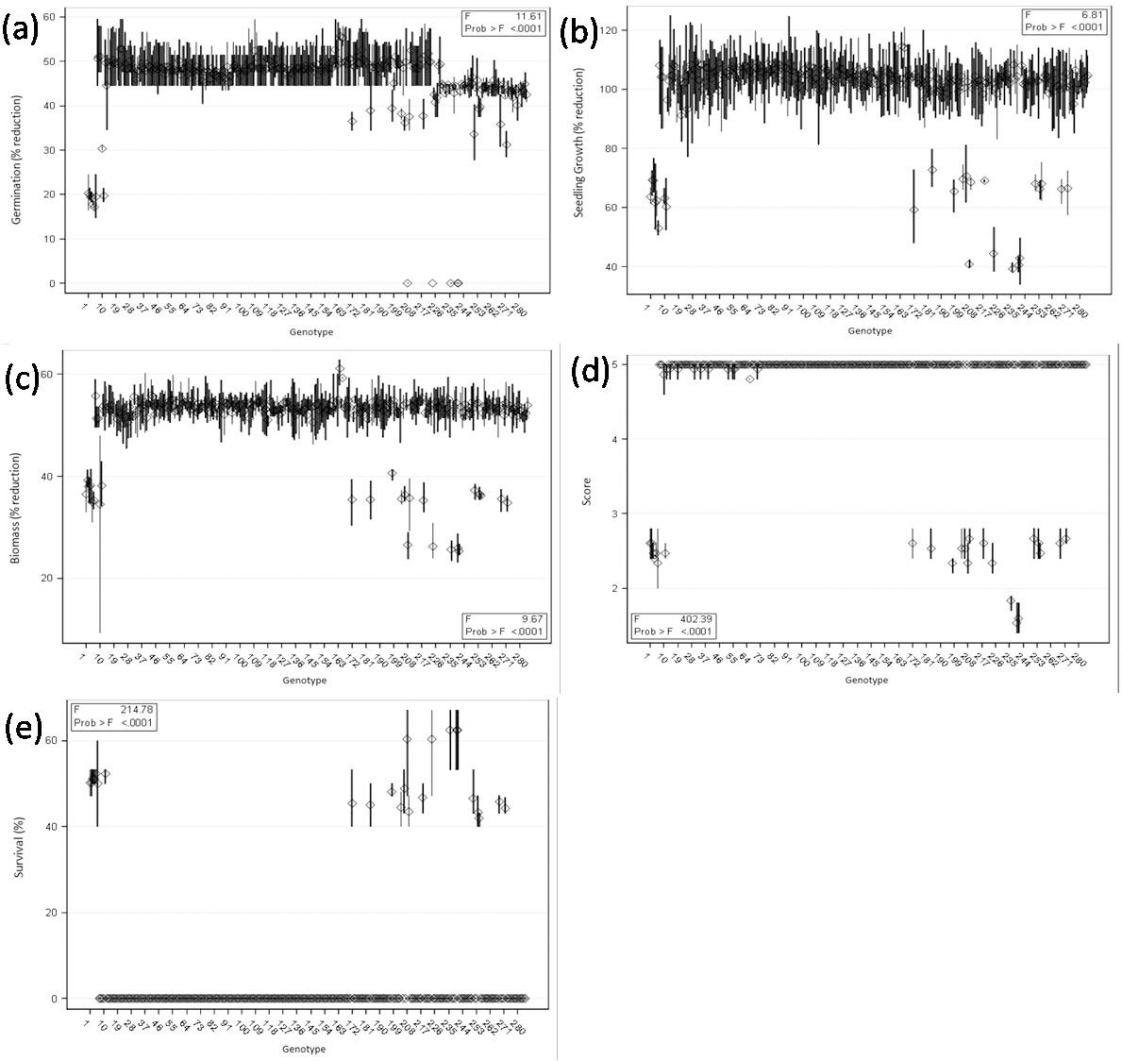
Effects of alkalinity stress (40 mM NaHCO_3_) on germination (a), seedling growth (b), biomass accumulation (c), alkalinity scores (d) and survival percent (e) of lentil genotypes under hydroponic condition.

**Fig 2:**
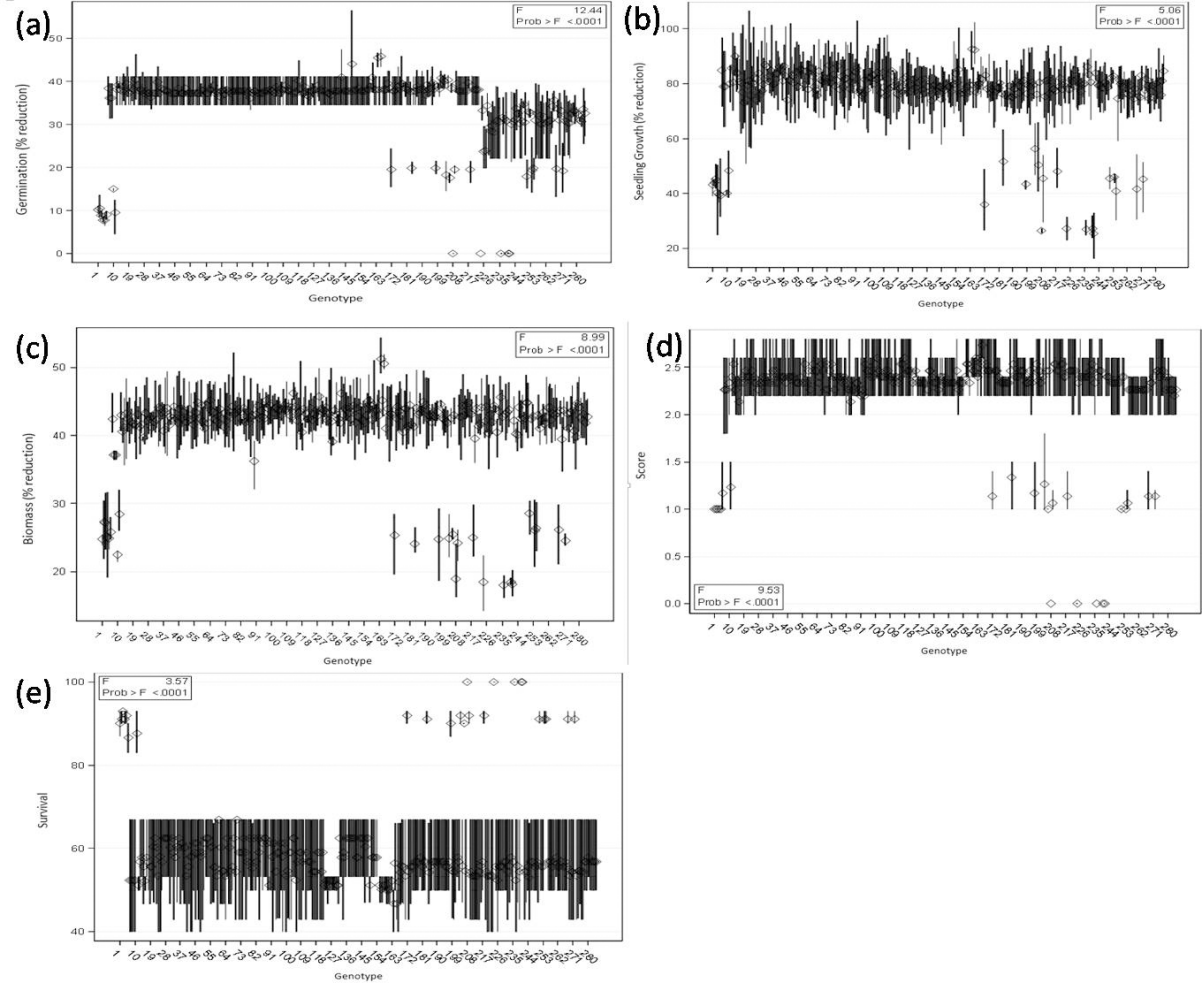
Effects of alkalinity stress (20 mM NaHCO_3_) on germination (a), seedling growth (b), biomass accumulation (c), alkalinity scores (d) and survival percent (e) of lentil genotypes under hydroponic condition.

**Fig 3:**
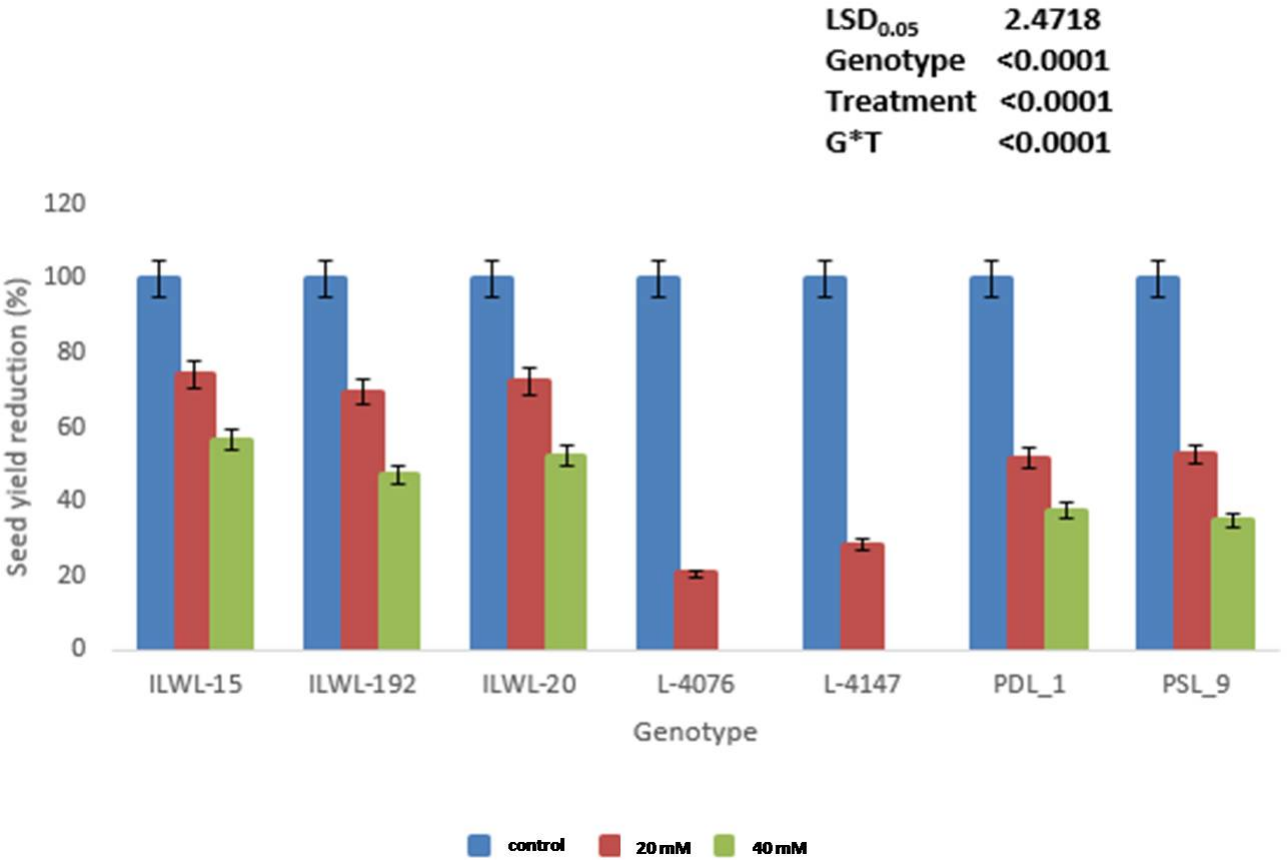
Reduction in seed yield plant^-1^ after exposure to 20 and 40 mM NaHCO_3_ at seedling stage in hydroponic condition.

The adverse effects of alkalinity stress on genotypes were found more pronounced at 40 mM NaHCO_3_ than at 20 mM NaHCO_3_ (Figs 1 and 2, S1 Table). However, there was no adverse effect on germination of wild accessions (ILWL-15, ILWL-20 and ILWL-192) and tolerant breeding lines (PDL-1 and PSL-9) at 40 mM NaHCO_3_. The most sensitive cultivars (L-4076 and L-4147) showed a maximum reduction in germination at a similar salt concentration (Fig 1A). There was a significant reduction in seedling growth and biomass accumulation at 40 mM NaHCO_3_. However, the reduction was lower in tolerant wild accessions and breeding lines than moderately tolerant and sensitive ones (Fig 1B and 1C). Tolerant genotypes could not exhibit wilting or any visible symptoms under alkalinity stress (20mM NaHCO_3_) up to 15 d, whereas moderately tolerant and sensitive genotypes exhibited varying degrees of wilting and leaf chlorosis with a score of 1.0 to 1.3 and 2.1 to 2.7, respectively (Fig 2). At 40mM NaHCO_3_, tolerant genotypes exhibited moderate salt injury compared to moderately tolerant and sensitive genotypes **(**Fig 1D**) (**S1 Fig**)**. Alkalinity stress of 20 and 40 mM NaHCO_3_ concentration affected the seedling survival differently in tolerant, moderately tolerant and sensitive genotypes. Five genotypes among 285, showed highest seedling survival at the end of the experiment (15d) under 40 mM NaHCO_3_. Tolerant breeding lines (PDL-1 and PSL-9) and wild accessions (ILWL-15, ILWL-192 and ILWL-20) exhibited maximum seedling survival. Moderately tolerant genotypes showed intermediate seedling survival, whereas the majority of the sensitive genotypes did not survive at a similar salt concentration (Fig 1E**)**. The survived tolerant genotypes exhibited large reduction in seed yield per plant at 40 mM NaHCO_3_ as compared to 20 NaHCO_3_ under hydroponic conditions (Fig 3).

**Fig 4:**
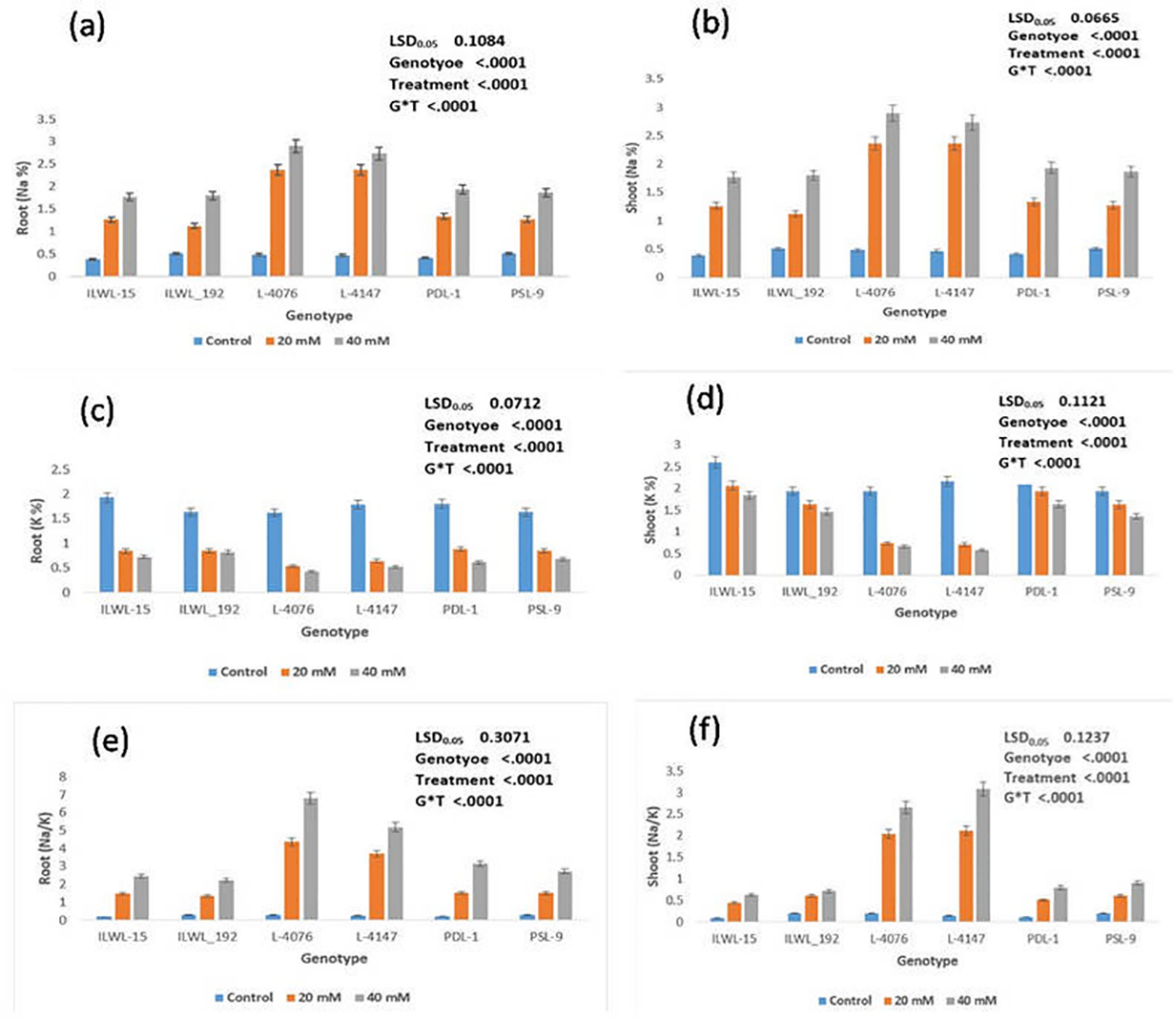
Effects of alkalinity stress (0, 20 and 40 mM NaHCO_3_) on Na^+^, K^+^ contents and Na^+^/K^+^ ratio in roots and shoots at seedling stage under hydroponic conditions.

Considerable differences in Na^+^ and K^+^ contents in both the roots and shoots were observed under alkalinity stress. It was evident that Na^+^ accumulated more in roots and shoots of sensitive genotypes than in tolerant ones at 20 mM and 40 mM NaHCO_3_ (Fig 4A and 4B). However, it is worth mentioning here that most sensitive cultivars (L-4076 and L-4147), accumulated the highest amount of Na^+^ in roots and shoots at 40 mM NaHCO_3_). The Na^+^ contents in roots and shoots were minimum in tolerant (PDL-1, PSL-9 ILWL-15, ILWL_192 and ILWL-20) genotypes at 40 mM NaHCO_3_. Potassium concentration declined considerably in both the tolerant and sensitive genotypes at both 20 and 40 mM bicarbonate (NaHCO_3_) concentration (Fig 4C and 4D). These results evidenced that the K^+^ concentration in roots and shoots was minimum in sensitive cultivars (L-4076 and L-4147), which was markedly affected by 40mM NaHCO_3_ under hydroponics. The similar trends of accumulation of Na^+^ and K^+^ in roots and shoots were also observed at 20 mM NaHCO_3._ The Na^+^/K^+^ ratio was higher in sensitive cultivars under the hydroponic condition (Fig 4E and 4F).

**Fig 5:**
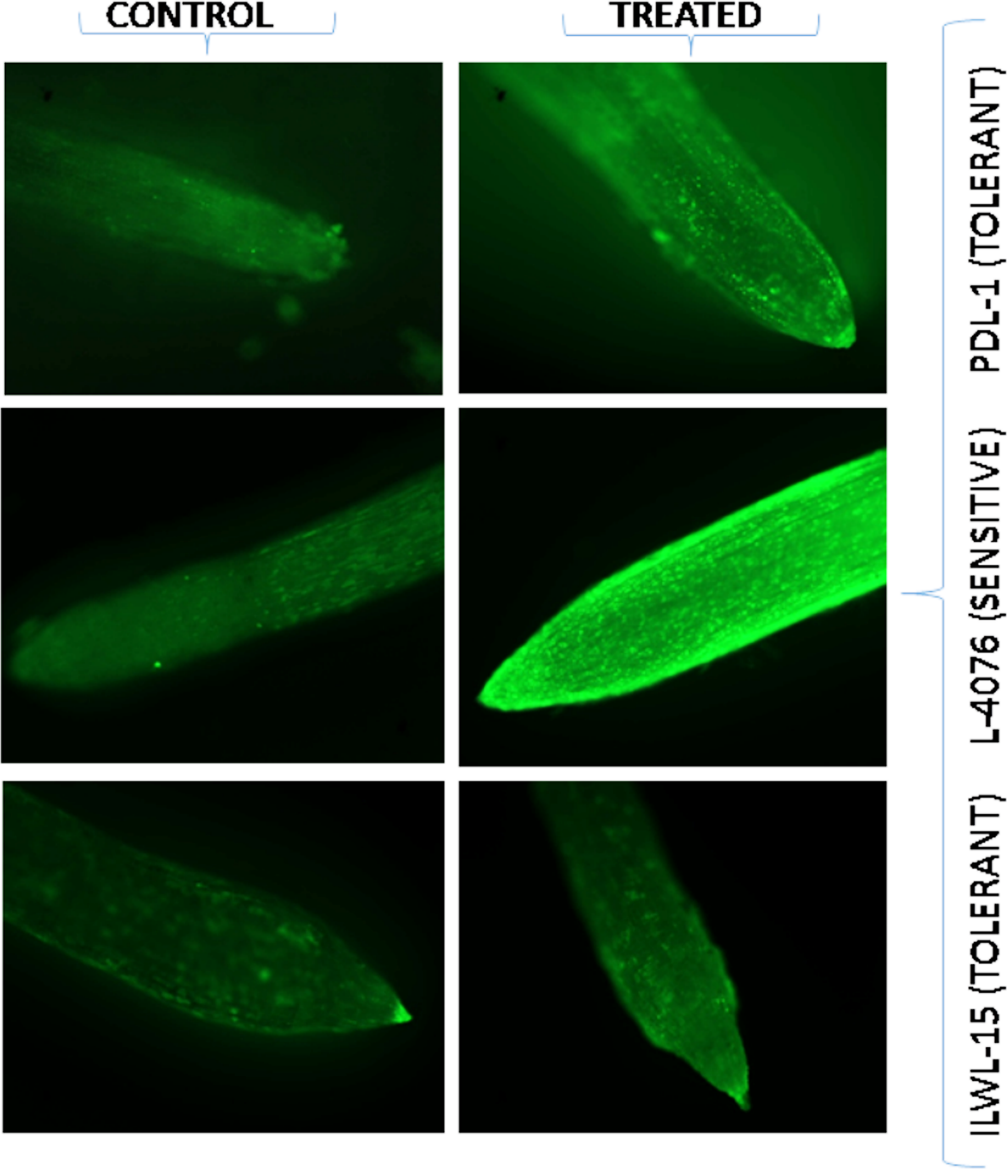
Detection of H_2_O_2_ by FDA in roots of tolerant breeding line (PDL-1), sensitive cultigen (L-4076) and tolerant wild accession (ILWL-15) under control and 40 mM NaHCO_3_ treatment condition.

Visualization of hydrogen peroxide (H_2_O_2_) production in roots using FDA produced green fluorescent in response to 40mM NaHCO_3_ treatment; the FDA fluorescence was negligible in the roots of control plants, whereas it increased markedly under 40mM NaHCO_3_. The level of H_2_O_2_ was higher in both the most tolerant and most sensitive genotypes under 40mM NaHCO_3_, when compared with their respective controls. Low fluorescent signals were observed in roots of the most tolerant breeding line (PDL-1), whereas intense green fluorescence was found in roots of most sensitive cultigen (L-4076) at 40 mM NaHCO_3_. Wild tolerant (ILWL-15) accession exhibited less green fluorescence when compared to tolerant breeding line (PDL-1), indicating less H_2_O_2_ production at the similar level of alkalinity stress (Fig 5).

**Fig 6:**
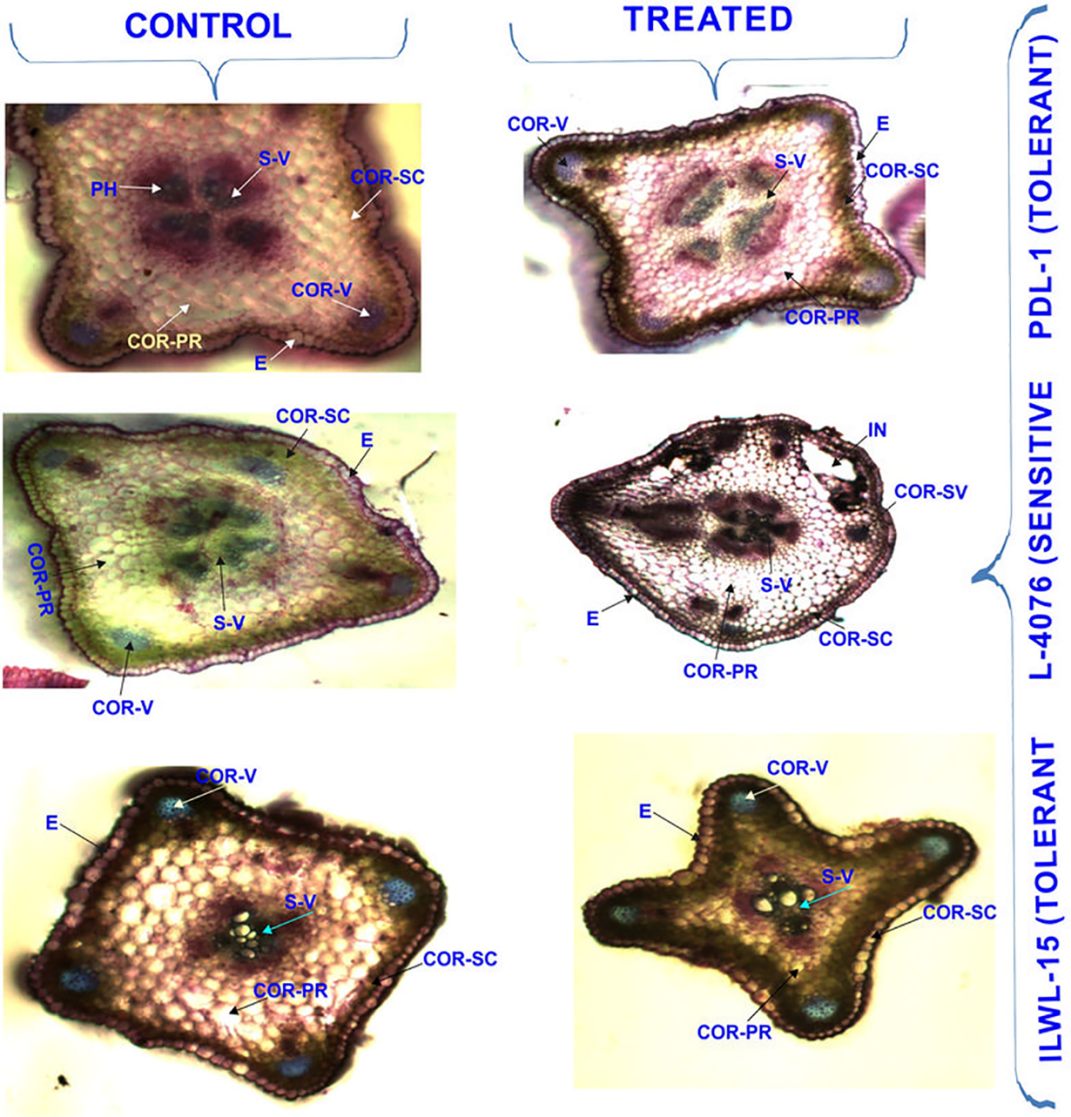
Cross-sections of stem of tolerant and sensitive genotypes under control (0 mM) and (40 mM NaHCO_3_) alkalinity stress. E-Epidermis, COR-SC—Cortical-Schlerenchyma, COR-PR- cortical parenchyma, PH = Phloem, END = Endodermis, S-V = Stellar-Vascular bundle, COR-V = Cortical -Vascular bundle.

**Fig 7:**
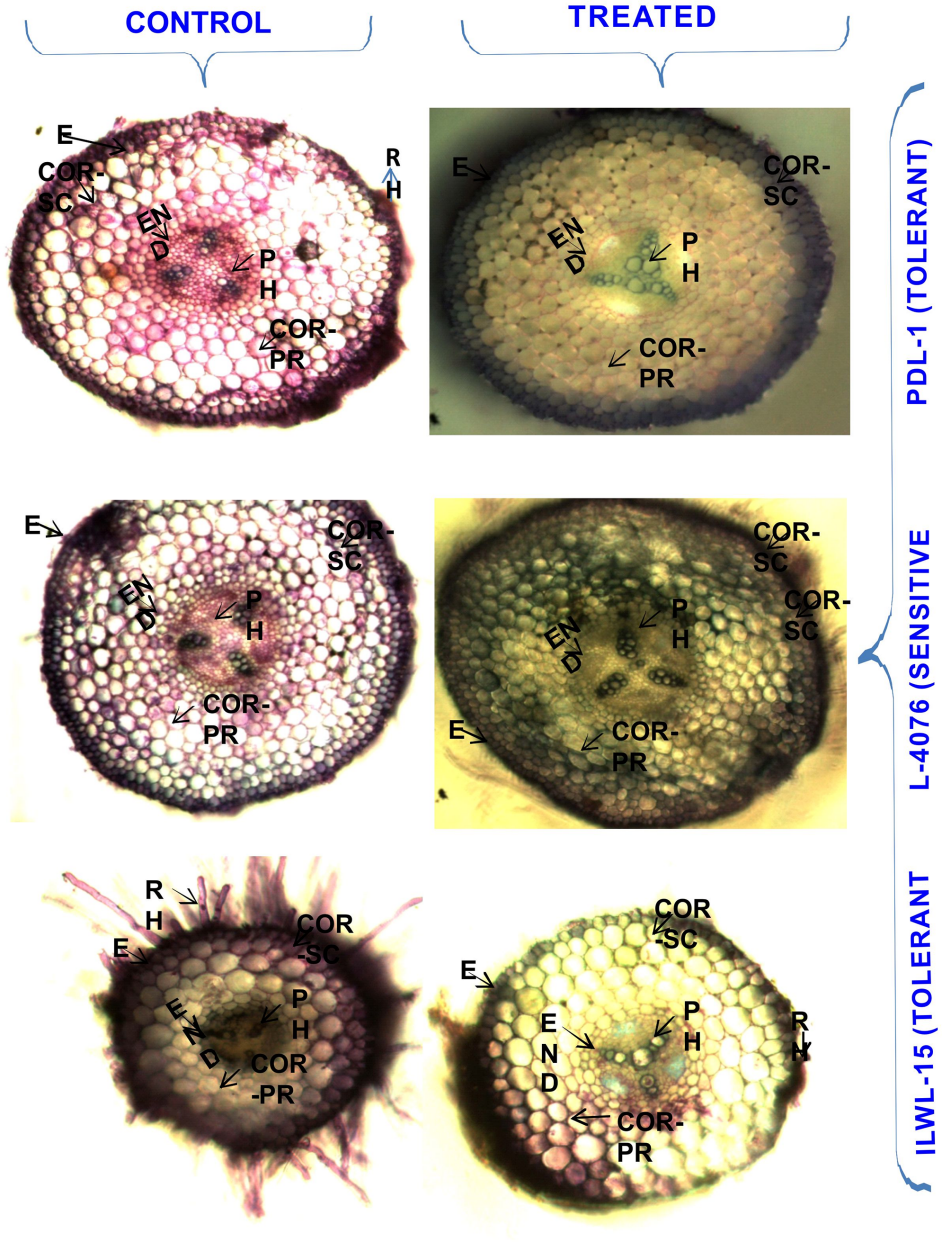
Cross-sections of root of tolerant and sensitive genotypes under control (0 mM) and (40 mM NaHCO_3_) alkalinity stress conditions. E = Epidermis, COR- SC = Cortical-Schlerenchyma, COR- PR = Cortical Parenchyma, PH = Phloem, RH = Root Hair.

Shoot anatomy of tolerant genotypes under alkalinity stress condition (40 mM NaCl) showed an enlarged pith area and increased vascular bundles and decreased cortical area. In sensitive cultivars, pith area was shrunken due to deformed stem structure. In stems, deposition was confined within the layers of cortical-sclerenchyma in tolerant breeding line (PDL-1). However, in sensitive cultivar (L-4076), deposition deeply penetrated many layers apart from cortical-sclerenchyma, including the cortical vascular bundles. Injury within the cortical layers of sensitive cultivar was noticed, whereas tolerant breeding line and wild accessions showed intact cortical region under the similar level of alkalinity stress (Fig 6). In roots, depositions were restricted to epidermis and cortical sclerenchyma only in both the tolerant breeding lines and wild accessions, whereas depositions spread all over the cortical region in the sensitive cultivar. Deformed epidermal and cortical cell structures were noticed under alkalinity stress conditions in sensitive cultivars in contrast to the tolerant breeding line, where the cells were intact but slightly enlarged as compared to its control (Fig 7).

### Potential components revealing alkalinity stress tolerance in the field

**Fig 8:**
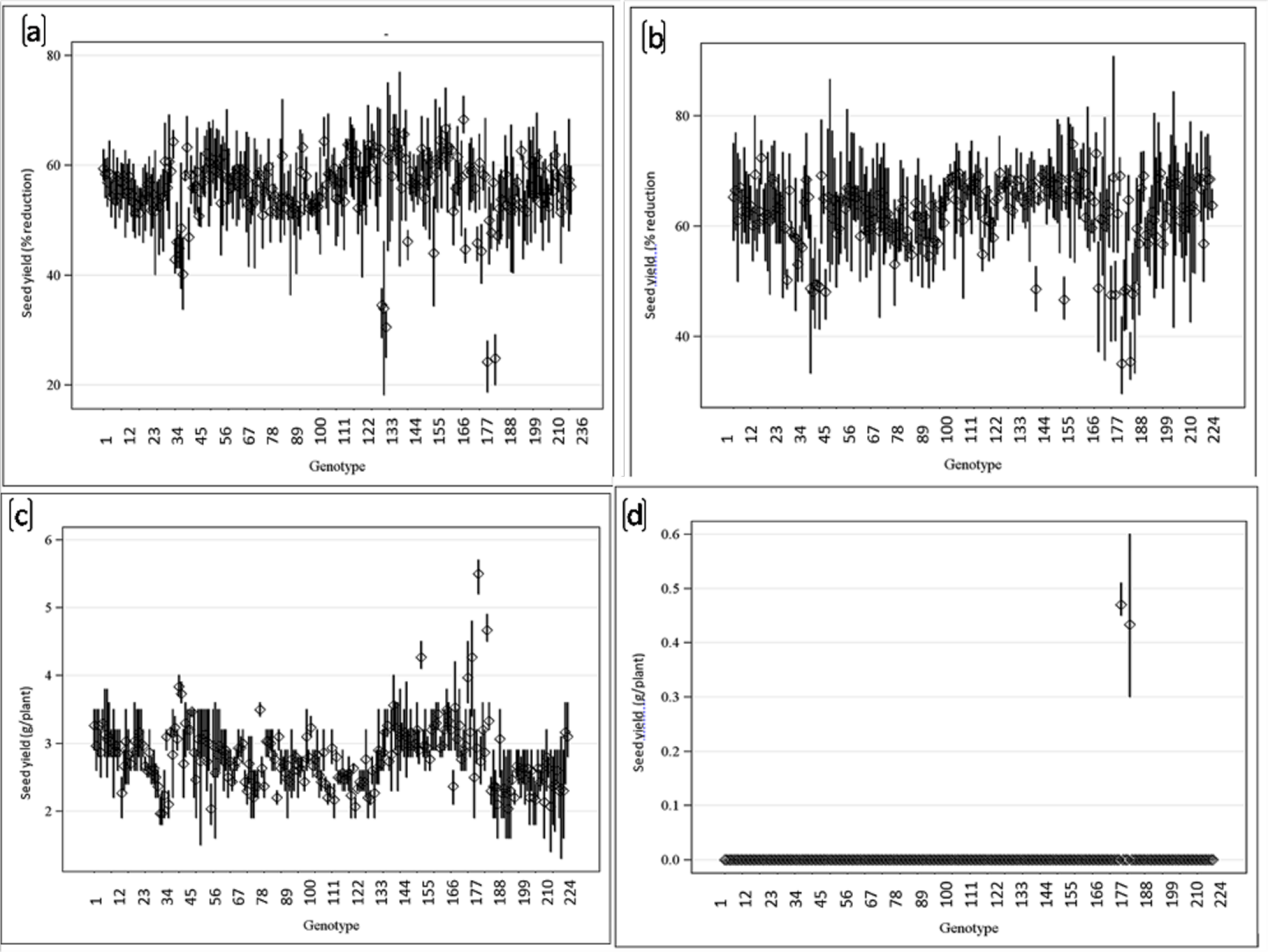
Seed yield reduction of genotypes grown under high alkalinity stress at Kanpur (a and b) pH (9.1) and Lucknow (c and d) under pH 9.0 and 9.5 during 2013–14 and 2014–15.

**Fig 9:**
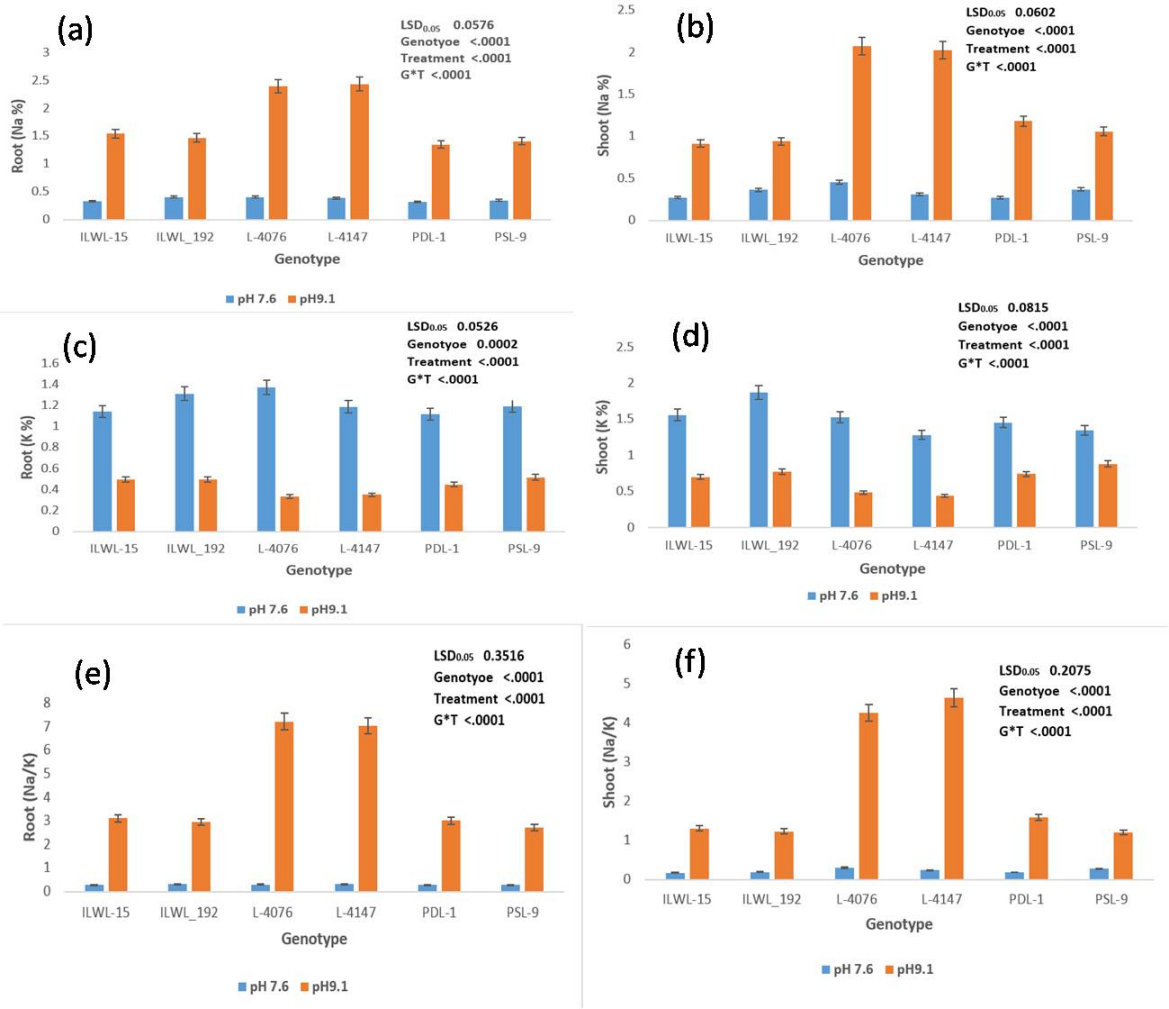
Effects of control (pH 7.6) and alkalinity stress (pH 9.1) on Na^+^, K^+^ contents and Na^+^/K^+^ ratio in roots and shoots at flowering stage under field condition.

Genotypes showed difference in seed yield at pH 9.0, 9.1 and 9.5 under field conditions. Reduction in seed yield under pH 9.1 over normal was observed in all the genotypes at Kanpur, India. However, the decline was much lower in tolerant breeding lines and wild accessions (PDL-1 PSL-9, ILWL-15 ILWL-20 and ILWL-192). The tolerant breeding lines PDL-1 and PSL-9 showed a minimum reduction in seed yield, while sensitive ones L-4076 and L-4147 exhibited maximum reduction at pH 9.1 (Fig 8A). The maximum seed yield/plant was obtained in tolerant breeding line at Lucknow under pH 9.0 (Fig 8C). Increasing the pH level beyond 9.0 showed detrimental effects on seed yield, where tolerant breeding lines PDL-1 and PSL-9 could produce negligible seed yield/plant, while sensitive cultivars did not produce any seeds (Fig 8D). Tolerant breeding lines (PDL-1 and PSL-9) and wild accessions (ILWL-192, ILWL-15 and ILWL-20) performed better than sensitive ones (L-4076 and L-4147) and the differences were quite obvious at seedling and reproductive stages at both the locations during 2013–14 and 2014–15. The tolerant breeding lines and wild accessions had much lower Na^+^ in roots and shoots^,^ and maintained relatively higher K^+^ uptake at pH 9.1, but the sensitive cultivars were unable to prevent either Na^+^ accumulation or K^+^ depletion under similar conditions (Fig 9A, 9B, 9C and 9D). Na^+^ contents in roots and shoots were higher at flowering stage than in vegetative stage (data not shown). Na^+^/K^+^ ratio was higher in sensitive cultivars than in tolerant ones under field conditions (Fig 9E and 9F).

### Relationship and ranking of genotypes grown in hydroponic and field screening methods

A relationship was studied between the two screening methods for 14 traits. The analysis gave r values ranging from 0.009 to 0.991 (Table 2). The hydroponic technique showed a significant correlation among the parameters such as reduction in germination, seedling growth, biomass accumulation, seed yield, seedling survival, alkalinity score, total sodium and potassium contents (hydroponic) and seed yield during 2013 and 2014 (field). The field screening technique exhibited significant correlation among all the morpho-physiological traits. Based on these results, the hydroponic method was adjudged to be the simple, rapid and reliable assay for screening of genotypes at the seedling stage for alkalinity tolerance.

**Table 2:** Correlation coefficient between reduction in seedling growth and biomass, seedling survival, alkaline scores, Na^+^, K^+^ and Na/K rat ratio attributes of alkalinity stress grown in the hydroponic at 40 mM NaHCO_3_ and in the field condition.

	RGM	RSG	R.BIOMASS	SS	SCORE	RSYH	H T Na^+^	H T K^+^	YLD 13	YLD 14	YLD 15	FTNa^+^	FTK^+^	FNa/K^+^	HNa/K^+^
RGM															
RSG	0.987**														
R.BIOMASS	0.991**	0.977**													
SS	0.974**	0.957**	0.968**												
SCORE	-0.976**	-0.970**	-0.968**	-0.977**											
RSYH	0.939**	0.945**	0.935**	0.923**	-0.965**										
H T Na^**+**^	-0.971**	-0.959**	-0.963**	-0.952**	0.971**	-0.946**									
H T K^**+**^	0.946**	0.961**	0.949**	0.934**	-0.951**	0.956**	-0.933**								
YLD 13	0.906**	0.889**	0.880**	0.866**	-0.847**	-0.792**	-0.832**	0.792**							
YLD 14	0.970**	0.963**	0.957**	0.965**	-0.975**	-0.957**	-0.960**	-0.939**	0.951**						
F TNa^+^	-0.109	-0.115	-0.080	-0.115	0.129	-0.131	0.045	-0.106	-0.190	-0.021	-0.062				
F TK^+^	0.114	0.080	0.099	0.109	-0.081	0.009	-0.052	-0.045	0.265	0.204	0.196	-0.932**			
F Na/K^+^	-0.360	-0.346	-0.331	-0.354	0.354	-0.323	0.285	-0.298	-0.434	-0.287	-0.313	0.285	-0.298**		
H Na/K^+^	-0.991**	-0.991**	-0.985**	-0.973**	0.984**	-0.958**	0.982**	-0.971**	-0.872**	-0.973**	-0.953**	0.982**	-0.971**	0.313	

RGM = Reduction germination; R.S.G = Reduction seedling growth; R. biomass = Reduction biomass; SS = Seedling survival; RSYH = Reduction seed yield hydroponic; HT Na^+^ = Hydroponic total sodium; HT K^+^ = Hydroponic total potassium; YLD 13 = Yield-2013; YLD14 = Yield 2014; YLD15 = Yield 2015; F TNa^+^ = Field total sodium; F TK^+^ = Field total potassium; F Na^+^/K^+^ = Field sodium, potassium ratio; H Na/K^+^ = Hydroponic sodium / potassium ratio.

** indicates (P<0.001).

The ranking of 236 genotypes in hydroponics was compared in respect of seed yield produced under field conditions during 2013–14 to evaluate the consistency in performance of genotypes. The ranking was based on most effective trait i.e. seedling survivability at 40 mM NaHCO_3_ and seed yield in the field and was found significantly correlated (r = 0.659; P = 0.0029). All the genotypes showed similar response under hydroponic and field conditions. For example, PDL-1, PSL-9, ILWL-15, ILWL-20 and ILWL-192 maintained alkalinity tolerance at both seedling and reproductive stages under both the techniques.

### Molecular marker analysis

A set of 495 primers which were pre-screened against most tolerant breeding lines (PDL-1 and PSL-9) and most sensitive cultivars (L-4147 and L-4076), from them 30 SSR primers, which exhibited polymorphism were selected for genetic diversity analysis among 285 genotypes. All the 30 SSR primer pairs generated polymorphic bands among the genotypes. A total of 146 alleles were identified with an average of 4.87 alleles per locus. The number of alleles per locus ranged from 3 (PBA_LC_1367, PBA_LC_1308, PBA_LC_404, PBA_LC_1241to 10 (LC_02). The major allele frequency varied between 0.26 (LC_02) to 0.77 (LC_39) with a mean value of 0.53. The gene diversity and PIC values varied between 0.37–0.81 and 0.34–0.79, with an average of 0.59 and 0.53, respectively. The primer which showed highest gene diversity and PIC values was LC_02, while the lowest gene diversity and PIC values were observed for the primer PLC_39. Heterozygosity in all the genotypes ranged from 0 to 0.30 with a mean value of 0.06 and the highest heterozygosity was observed in LC_02 (S2 Table).

By using 38 additional genomic SSR markers PIC and genetic diversity remained similar but the heterozygosity level increased (S2 Fig and S3 Table).

#### Cluster analysis of morpho-physiological traits

**Fig 10:**
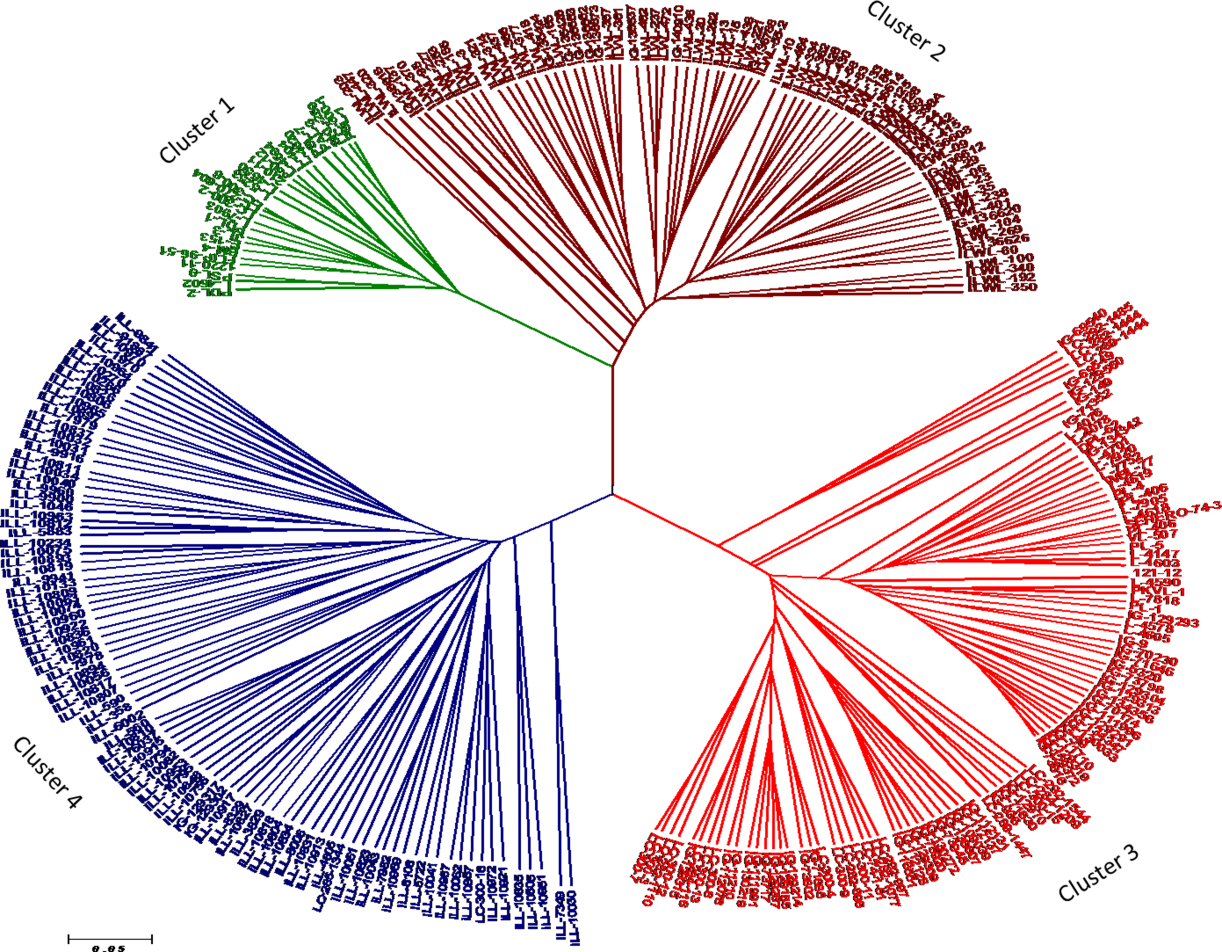
UPGMA tree based on dissimilarity index of 30 SSR markers for 285 lentil genotypes.

The genetic relationships among lentil genotypes are presented in SSR based UPGMA tree (Fig 10). All the genotypes were grouped into four clusters using 30 polymorphic SSR markers. Tolerant breeding lines fell with cluster C1, while sensitive ones in C3. Cluster 2 included wild accessions. Cluster 4 consisted of 90 genotypes, which grouped the “ILL” series of genotypes mainly. The mean genetic distance of the clusters ranged from 0.57 to 0.61 with an average of 0.58. Cluster 4 showed the highest mean genetic distance of 0.61, followed by clusters 2, 3 and 1 (Table 3). The average reduction percent in germination, root and shoot length, fresh and dry root and shoot weight, seedling survivability and alkalinity scores were calculated among the clusters categorized by SSR markers of 285 genotypes. Among the SSR clusters, there was a wide range in the values for most of the characters analyzed. Significant (P = 0.05) differences for all the characters were observed among the clusters. There were significant differences between the parameter for the genotypes of clusters 1 and 2 which comprised of cultivars, breeding lines and wild accessions, as compared to those of other clusters (Table 3). The lowest alkalinity score (4.19), reduction in root length (40.45%), shoot length (49.61%), fresh and dry root weight (40.37%, 41.67%) and shoot weight (47.32%, 58.58%) was observed in the tolerant genotypes of cluster 1 followed by cluster 2. There was no significant difference between the parameter of clusters 3 and 4 (Table 3). These differences in the growth parameters may be due to higher alkalinity tolerance among cultivars of cluster 1 and wild accessions of cluster 2. The clusters based on SSR markers have been found to have a relationship with the degree of alkalinity tolerance. The wild accessions grouped in cluster 2 showed greater proximity with cluster 1 with a similar degree of alkalinity stress tolerance. Whereas, 38 additional SSR markers used in this study, a total of six clusters were observed. This clustering pattern did not show a clear relationship between clusters and degree of alkalinity tolerance in most genotypes with respect to different traits used in the present study (S4 Table).

**Table 3:** Cluster mean for reduction in germination, root and shoot length, fresh and dry weight of roots and shoots, seedling survival% and mean genetic distance (MGD) under 40mM NaHCO_3_.

Cluster	GM	RL	SL	FWR	FWS	DWR	DWS	survival	Score	MGD
cluster 1	40.04^a^	40.45^a^	49.61^a^	40.37^a^	47.32^a^	41.67^a^	58.58^a^	16.61^a^	4.19^c^	0.57
cluster 2	42.52^b^	44.40^b^	53.74^b^	43.16^b^	53.34^b^	45.01^b^	62.71^b^	5.45^b^	4.71^b^	0.58
cluster 3	48.94^c^	46.24^c^	55.76^c^	47.34^c^	53.17^b^	46.87^c^	65.25^c^	1.55^c^	4.92^a^	0.57
cluster 4	47.05^c^	47.03^c^	55.83^c^	46.05^c^	52.88^b^	46.87^c^	65.27^c^	1.73^c^	4.90^a^	0.61

GM-germination; RL-root length; SL-shoot length; FWR-fresh weight root; FWS-fresh weight shoot; DWR-dry weight root; DWS-dry weight shoot; MGD-mean genetic distance

Values within each column that do not share common letter are significantly different by Duncan’s post- hoc test at P≤0.05.

**Fig 11:**
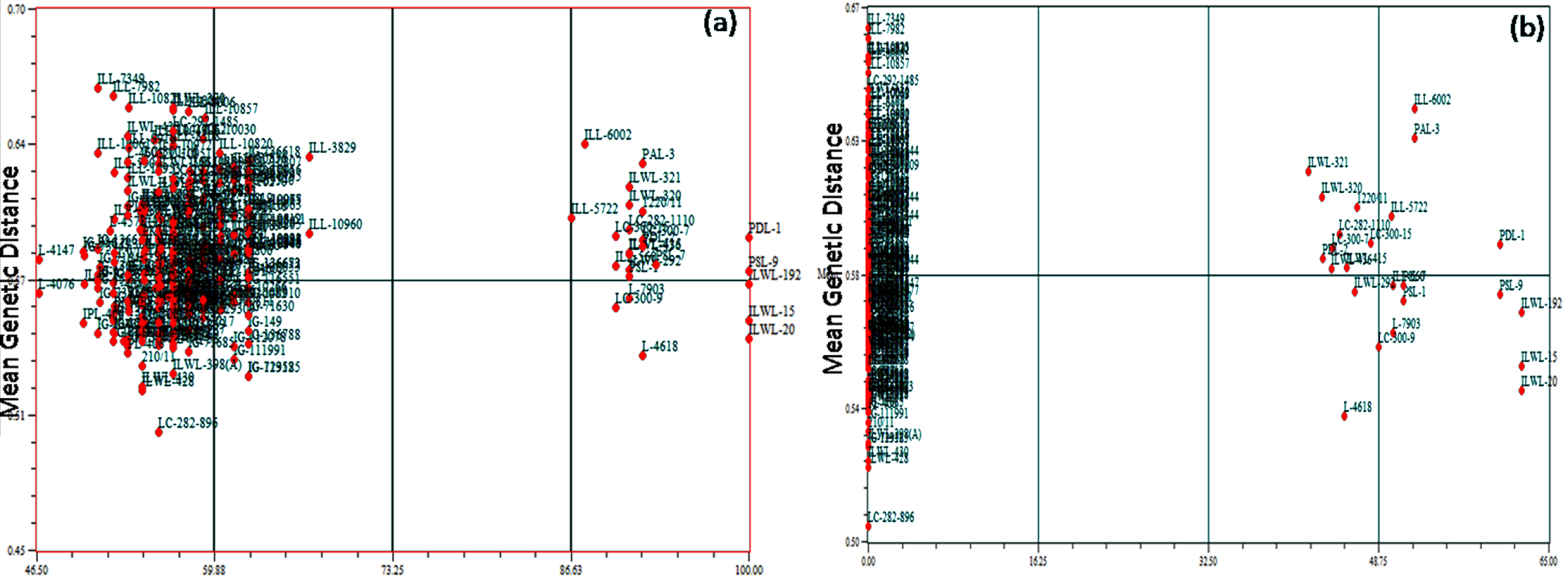
Correlation between genetic similarity index and taxonomic distance for seedling survivability percent of 285 genotypes at 20 and 40mM NaHCO_3_ concentrations.

Further, tolerant and sensitive genotypes were separated when the correlation between genetic similarity index and taxonomic distance for seedling survivability percentage was evaluated using Jaccard similarity index under both the alkaline stress conditions (Fig 11A and 11B). Sensitive genotypes showed no seedling survivability, whereas tolerant ones exhibited 40% seedling survivability under 40mM M NaHCO_3_ concentration. However, similar results were obtained with respect to 30 and 68 SSR markers during Jaccard’s similarity coefficient analysis between mean genetic distance and seedling survivability at both 20mM and 40mM concentrations, respectively (S4 Fig).

#### Population structure and genetic relationship among genotypes

**Fig 12:**
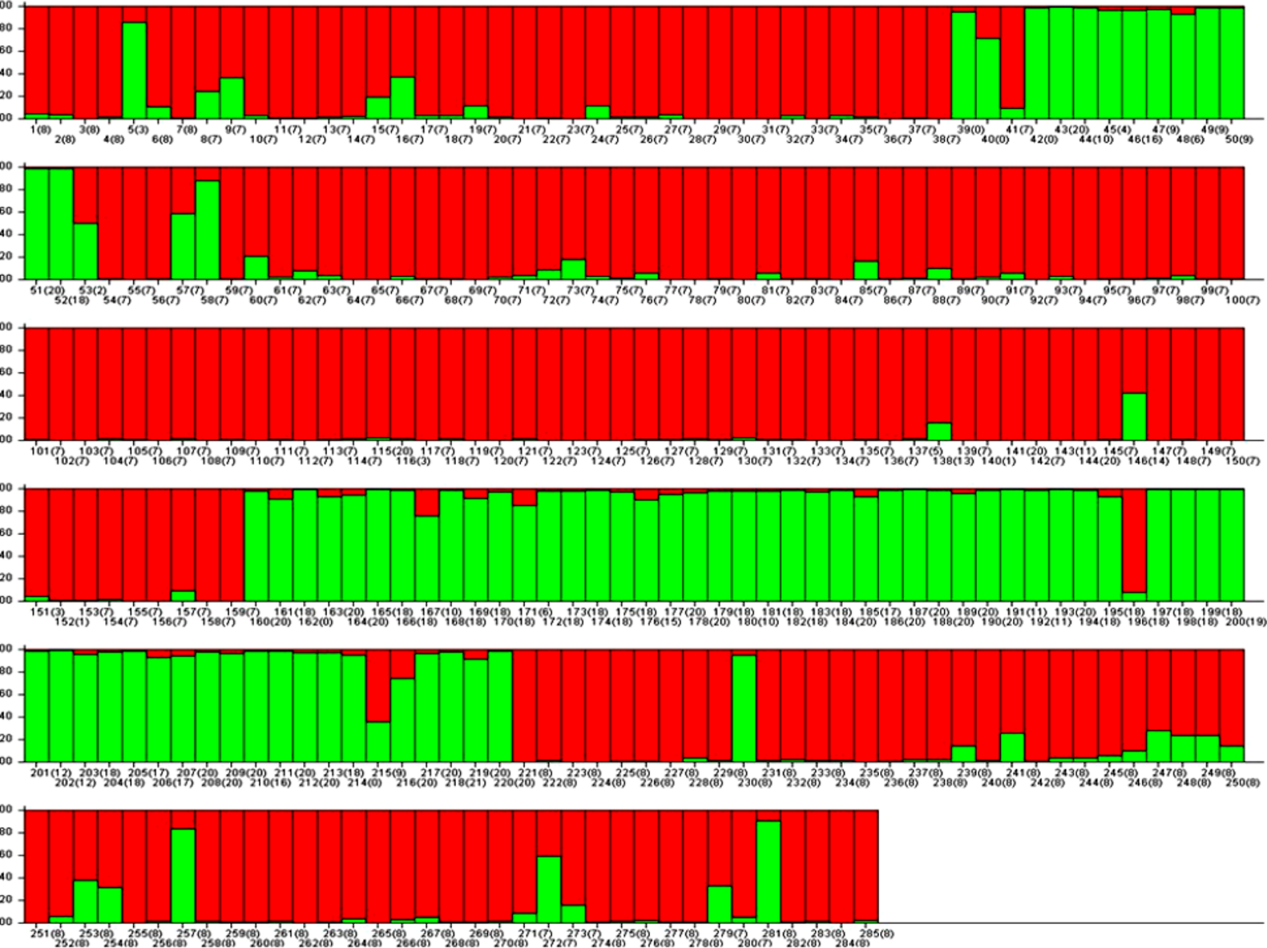
Structure plot with K = 2 depicting model based population, using structure with 30 SSR markers.

Pritchard’s structure of 285 *Lens* genotypes was estimated from SSR allelic diversity data, where the best goodness of fit was found at K = 2 (Fig 12). Using a membership probability threshold of 0.80, 195 genotypes were assigned to SG 1 (red), 74 genotypes were assigned to SG 2 (green), and 16 genotypes were in admixtures (AD) for 30 makers. The population structure was found mostly co-related to cluster data, as the two populations also separated most of the cultivars from wilds and ‘ILL’ series of cultivars. Alkalinity tolerant genotypes such as PDL-1, PSL-9 and ILWL-15 were found to have admixes but the inclination was towards SG 2 (green) whereas, sensitive ones such as L-4147 and L-4076 was confined to SG 2 (red) without showing any admixes.

Comparatively, 38 more number of arbitrary SSR markers, K = 3 was observed. It resulted in SG 1 (red) 127 genotypes, SG 2 (green) 49 genotypes, SG 3 (blue) 41 genotypes and 68 genotypes were in admixtures (AD) (S3 Fig)

## Discussion

Evaluation of germplasm to locate alkalinity tolerance gene (s) is important for incorporating the tolerance in high yielding cultivars. This requires the most effective screening system which allows the evaluation of genotypes for identification of alkaline tolerance. For identifying the genotypes for alkaline stress tolerance, hydroponic technique is an efficient tool where efficiency has already been established for other crop plants also [12, 32]. However, initial screening under hydroponics and later in field conditions would be the proper strategy. In the present work, a large number (285) of germplasm including cultivars, breeding lines, land races and wild accessions were evaluated based on morpho-physiological and anatomical traits which represent most of the possible variability available for use in breeding programs for alkalinity stress tolerance. Seed germination and early seedling growth are critical stages under salinity and alkalinity stresses [33]. In the present study, germination in all the genotypes except tolerant ones decreased at 40 mM NaHCO_3_ (Fig 1A). This might be because of an adverse effect of salt on water imbibitions by seed and toxic effect of ions on the seed metabolism. This result is an agreement with de Lacerdo et al. [34]. The tolerant wild accessions (ILWL-15, ILWL-192 and ILWL-20) showed no wilting but low salt injury, whereas tolerant lines and sensitive genotypes showed partial wilting with minimum and maximum score of salt injury at 40 mM NaHCO_3_, respectively (Fig 1B). Salt injury symptoms were found well correlated with seedling survival under alkalinity condition (r = -0.977**). This trait has been widely used parameter for selecting salt-tolerant lines and cultivars [35]. Breeding for seedling survival and vigorous growth has been argued to be agronomically most effective approach for increasing yield under saline soils [36]. Alkalinity stress at 40 mM NaHCO_3_ had affected the seedling survival in all the genotypes. However, tolerant breeding lines and wild accessions showed higher seedling survival than sensitive ones, which have no seedling survival under 40 mM NaHCO_3_ stress (Fig 1E). This study showed that plant survival was more powerful tool to characterize alkaline stress tolerance at seedling stage. These results are in concurrence with those of Murillo-Amador et al. [37]. Decreasing seedling survival under high concentration of NaHCO_3_ was also observed in seedling growth and biomass production. However, the extent of reduction was variable and less pronounced in tolerant genotypes than sensitive ones (Fig 1B and 1C).The inhibition in these traits might be largely due to the generation of ROS [38]. In the present study, the level of ROS induced H_2_O_2_ production as florescent imaging was found low in tolerant genotypes than in sensitive ones (Fig 5).

In root, thick endoderm and viable vascular bundles restrict the entry of Na^+^ in tolerant wild and breeding lines over the sensitive ones (Figs 6 and 7).The uptake of Na^+^ was also restricted by sclerenchymatous layers in tolerant breeding and wild accessions. Intact pericycle and steler region of tolerant genotypes also helped in reducing the ion absorption. Similar observations were also made in lentil [36], finger millet [23] and *Lotus tenuis* [11]. Similarly, the shoot had thick epidermis in tolerant genotypes compared sensitive ones. It helped in maintaining cell turgor pressure in tolerant lines, whereas in sensitive ones, thin and injured epidermis was noticed which allowed higher seepage of ions. Steler as well as cortical vascular bundles were found distorted in the sensitive genotypes which disturb the supply of food and water throughout the plant.

Based on 2 years seed yield data from two locations, tolerant breeding lines (PDL-1 and PSL-9) and wild accessions (IlWL-192, ILWL-15 and ILWL-20) ranked first with minimum reduction and L-4147 and L-4076 sensitive cultivars showed a higher reduction at pH 9.1. However, most tolerant breeding lines could withstand alkalinity stress up to pH 9.5, but gave negligible seed yield. The sensitive and moderately tolerant genotypes could not reach up to reproductive stage under high alkaline stress (pH 9.5) and therefore, showed 100% loss of seed yield (Fig 8D).This suggests that the best pH for selection for tolerance ranges between pH 9.0 and 9.1. Further, the range of variation for seed yield decreased with the increase in soil pH, suggesting the existence of narrow range of variability (Fig 8).The alkalinity tolerance in breeding lines and wild accessions showing low reduction in seed yield was found associated with lower Na^+^ accumulation and higher K^+^ and low Na^+^/K^+^ ratio as compared to the sensitive cultivars (Fig 9A, 9B, 9C and 9D).This suggested that regulation of Na^+^ uptake in tolerant breeding lines and wild accessions was accompanied by maintaining higher K^+^ which appeared to be the main factor responsible for better performance up to pH 9.1. Earlier studies have shown that tolerant genotypes maintain better regulation over Na^+^ and K^+^ uptake than sensitive ones [39, 40].

In order to assess the consistency in alkaline stress tolerance for ranking of genotypes both under hydroponics and field conditions, 236 genotypes were compared with seed yield under field condition during 2013–14. The ranking of genotypes based on seedling survivability in hydroponics at 40 mM NaHCO_3_ and seed yield in the alkaline field during 2013–14 (r = 0.659; P = 0.0029) was found significantly correlated. This consistency in ranking between hydroponics and field supports the reliability of the results and suggests that hydroponic screening is reliable technique for identifying alkalinity tolerant genotypes. To establish this fact the correlation coefficient between the seed yield and each of seedling growth, biomass, seedling survivability, alkalinity scores and Na^+^ and K^+^ and Na^+^/K^+^ ratio estimated (Table 2). The seed yield in the field was found highly correlated with seedling survivability under hydroponics. Thus, while screening a large number of genotypes for alkalinity tolerance, the selection of genotypes should be made on the basis of seedling survivability. Previous findings also provide support for the selection of alkaline tolerant genotypes on the basis of use of seedling survival. Murillo-Amador et al. [41] also suggested this trait on the basis of results obtained in cowpea.

The findings of the hydroponic method were found to be well correlated with field experiment. Similar result of genotypes for salinity tolerance in faba bean was reported by Tavakkoli et al [42]. The tolerant genotypes selected at the seedling stage in hydroponic, maintained their ranking for tolerance up to the adult stage under alkalinity field, suggesting that screening of the germplasm for alkalinity stress tolerance at the seedling stage is more effective under hydroponics.

All 285 lentil genotypes were also scanned with 30 SSR markers which were found polymorphic. The average number of the allele was comparable to the result of Dikshit et al. [43] using various *Lens* species and observed an average of 4.8 alleles per locus (range of 3 to 10. Similarly, Idrissi et al. [44], also reported an average of 5 alleles per locus using different lentil accessions. These differences in alleles per locus might be due to the genotypes used and selection of SSR markers. Markers that have the ability to detect high number of discernible alleles are the suitable markers for molecular characterization and genetic diversity analysis [44]. The results of this study suggest that these markers could be used as tools to assess the genetic diversity of lentil germplasm of various traits linked to alkalinity tolerance.

Polymorphic information content (PIC) value ranged from 0.34 to 0.79 with an average of 0.54 (S2 Table). The PIC values observed in this analysis were consisted with the previous report involved in microsatellite marker analysis in lentil. PIC value of this study was higher than the earlier reports which might be due to the inclusion of more diverse set of lentil germplasm. The PIC value of a marker reflects marker allele diversity and frequency among the cultivars. Higher the PIC value of a marker indicates a higher probability of detecting the number of alleles among cultivars. LC-02 had the highest PIC value (0.789), followed by PLC-100 (0.733) and markers which showed lowest PIC were PBA-LC-376 (0.349) followed by PLC-39 (0.342). Therefore, SSR marker, LC-02 was found to be superior for the analysis of genetic diversity in future. These findings describe the usefulness of these markers for characterizing lentil genotypes.

It was evident that better performance of tolerant wild accessions (ILWL-15, ILWL-192 and ILWL-20) and tolerant breeding lines (PDL-1 and PSL-9) under alkalinity stress was probably due to their higher seedling survivability, lesser reduction in seedling growth, biomass and seed yield, regulated the Na^+^ contents, low H_2_O_2_ production and a more uptake of K^+^. Five genotypes produced highest and most consistent seed yield in all the locations (across years). The wild lentil accessions (ILWL-15, ILWL-192 and ILWL-20) showed relatively higher level of tolerance and thus can be used for incorporating genes to improve alkalinity stress tolerance of the cultivated one either through conventional breeding or through genetic engineering. The lentil accessions at the extreme clusters can be used in breeding program to develop new tolerant genotypes for growing in alkaline soils. Although, more SSR markers were included in this study, but no clear association between clustering pattern and degree of alkalinity tolerance in most of the genotypes was found. Further work is needed to phenotype and genotype the RILs under alkaline and non-alkaline conditions, and to identify gene (s) for alkaline stress tolerance in lentil using marker-assisted selection.

## Supporting information

S1 Fig Phenotypic response of tolerant and sensitive genotypes under 40mM NaHCO_3_ concentration. (TIFF)

S2 Fig UPGMA tree based on dissimilarity index of 68 SSR markers for 285 lentil genotypes. (TIF)

S3 Fig Structure plot with K = 3 depicting model based population, using structure with 68 SSR markers. (JPG)

S4 Fig Correlation between genetic similarity index and taxonomic distance for seedling survivability percent of 285 genotypes at 20 and 40mM NaHCO_3_ concentrations. (TIF)

S1 Table Phenotypic responses of lentil genotypes under 20 and 40mM NaHCO_3_ concentrations. (XLSX)

S2 Table Allelic variations and PIC values for 30 SSR markers identified in 285 lentil genotypes. (DOCX)

S3 Table Allelic variations and PIC values for 68 SSR markers identified in 285 lentil genotypes. (DOCX)

S4 Table Cluster mean for reduction in germination, root and shoot length, fresh and dry weight of roots and shoots, seedling survival% and mean genetic distance (MGD) under 40mM NaHCO_3_. (DOCX)
