# Supplementary figures and images for: Evaluation of cultivated and wild genotypes of Lens species under alkalinity stress and their molecular collocation using microsatellite markers
Source: PLoS One. 2018 Aug 13;13(8):e0199933. doi: 10.1371/journal.pone.0199933 (PMC6089424; doi:10.1371/journal.pone.0199933)

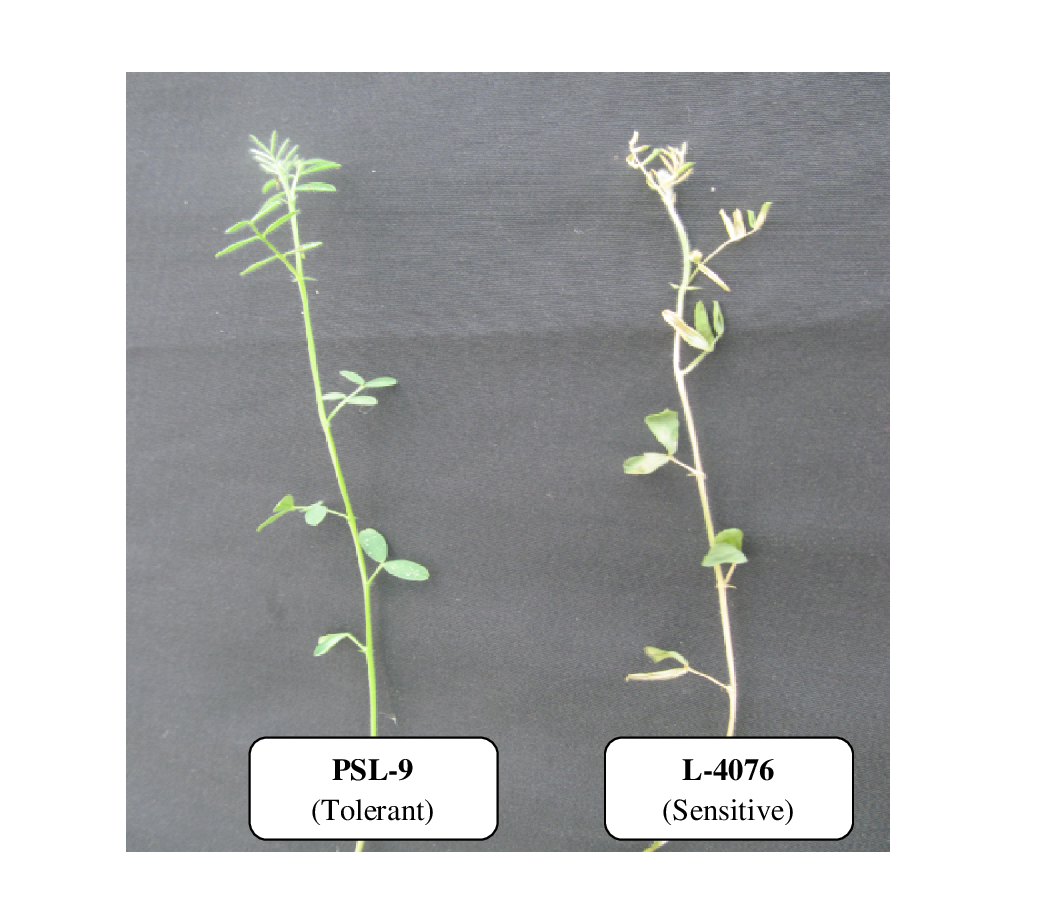

Supplement: S1 Fig — (TIFF) [file pone.0199933.s001.tiff]

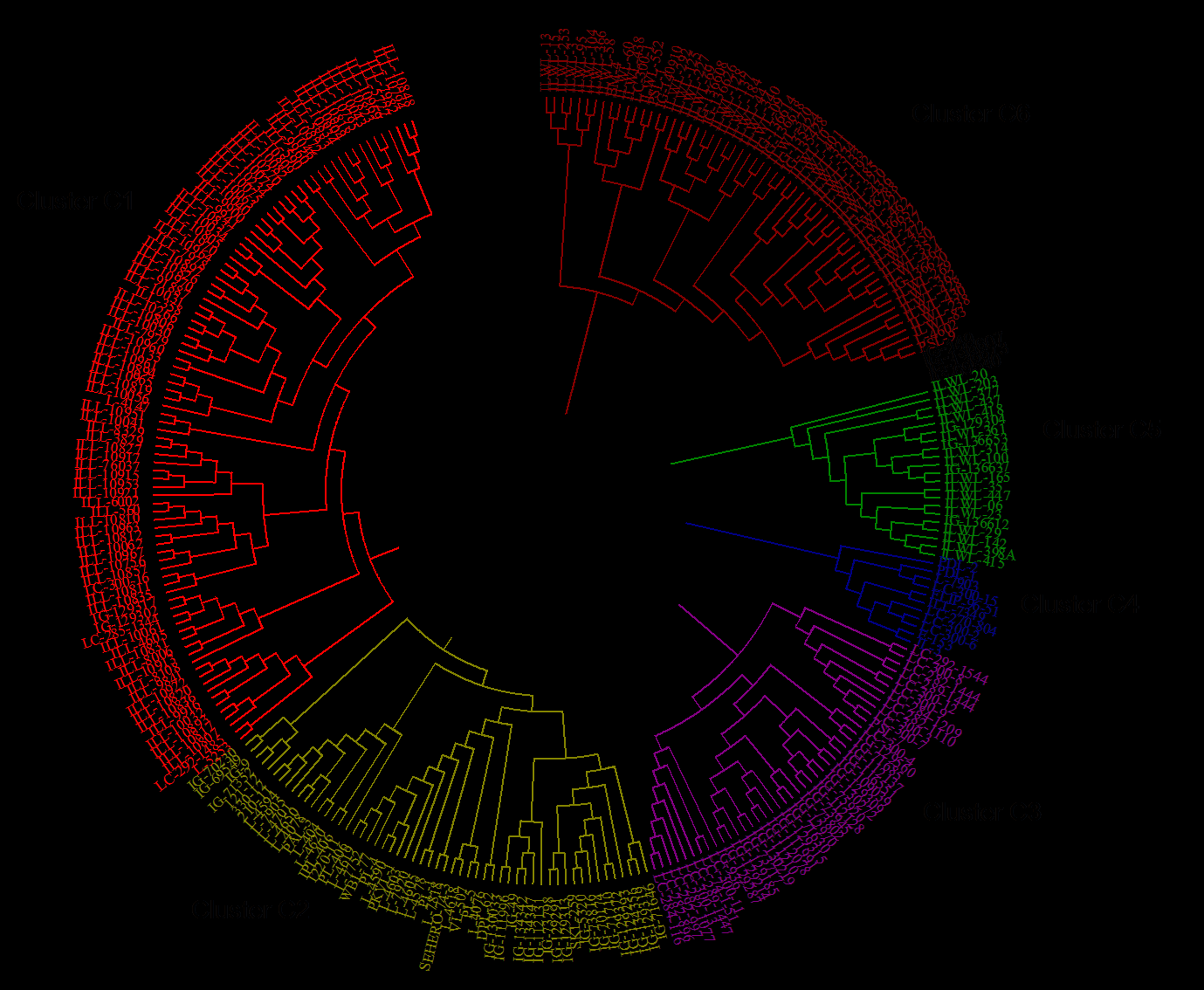

Supplement: S2 Fig — (TIF) [file pone.0199933.s002.tif]

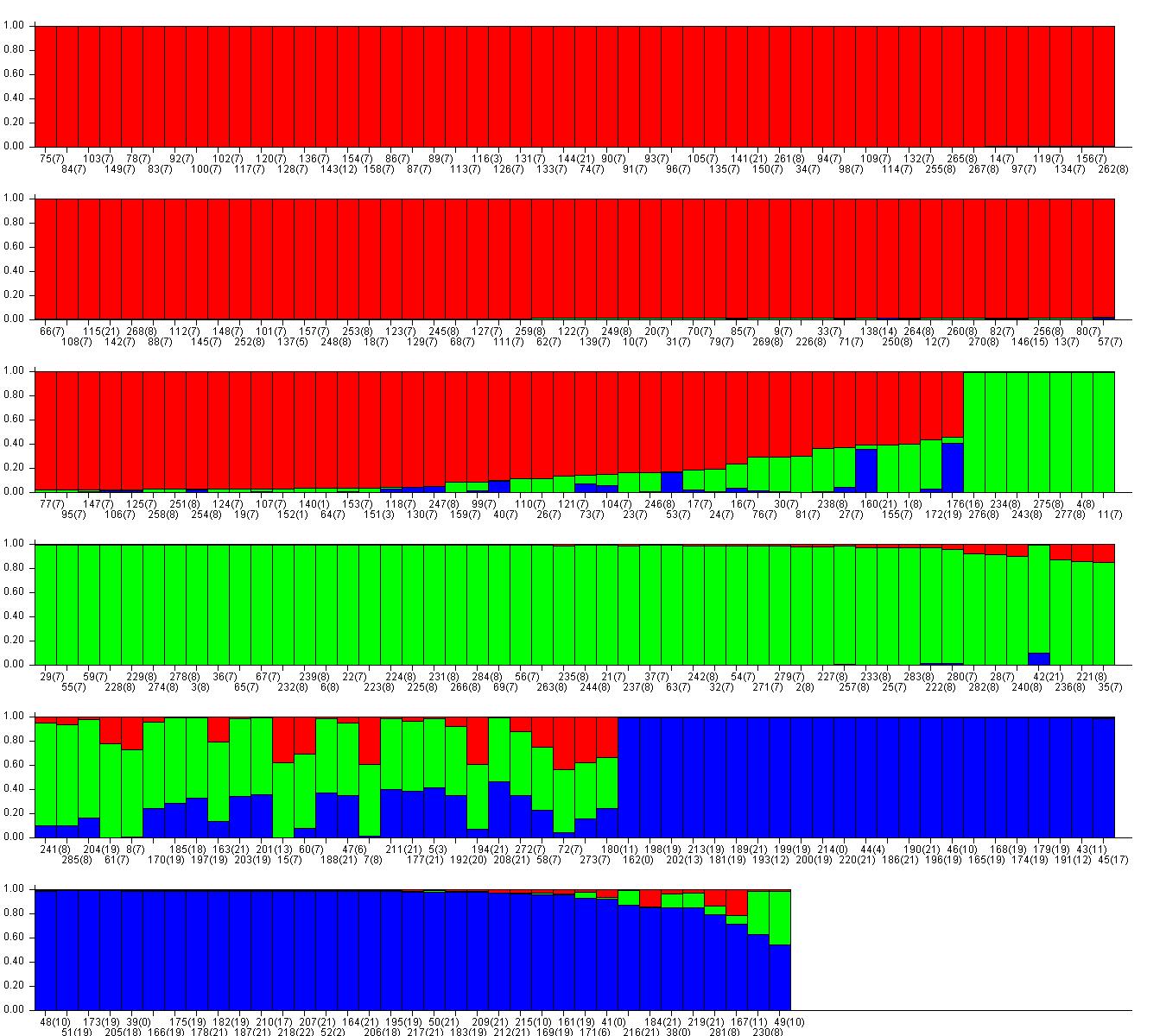

Supplement: S3 Fig — (JPG) [file pone.0199933.s003.jpg]

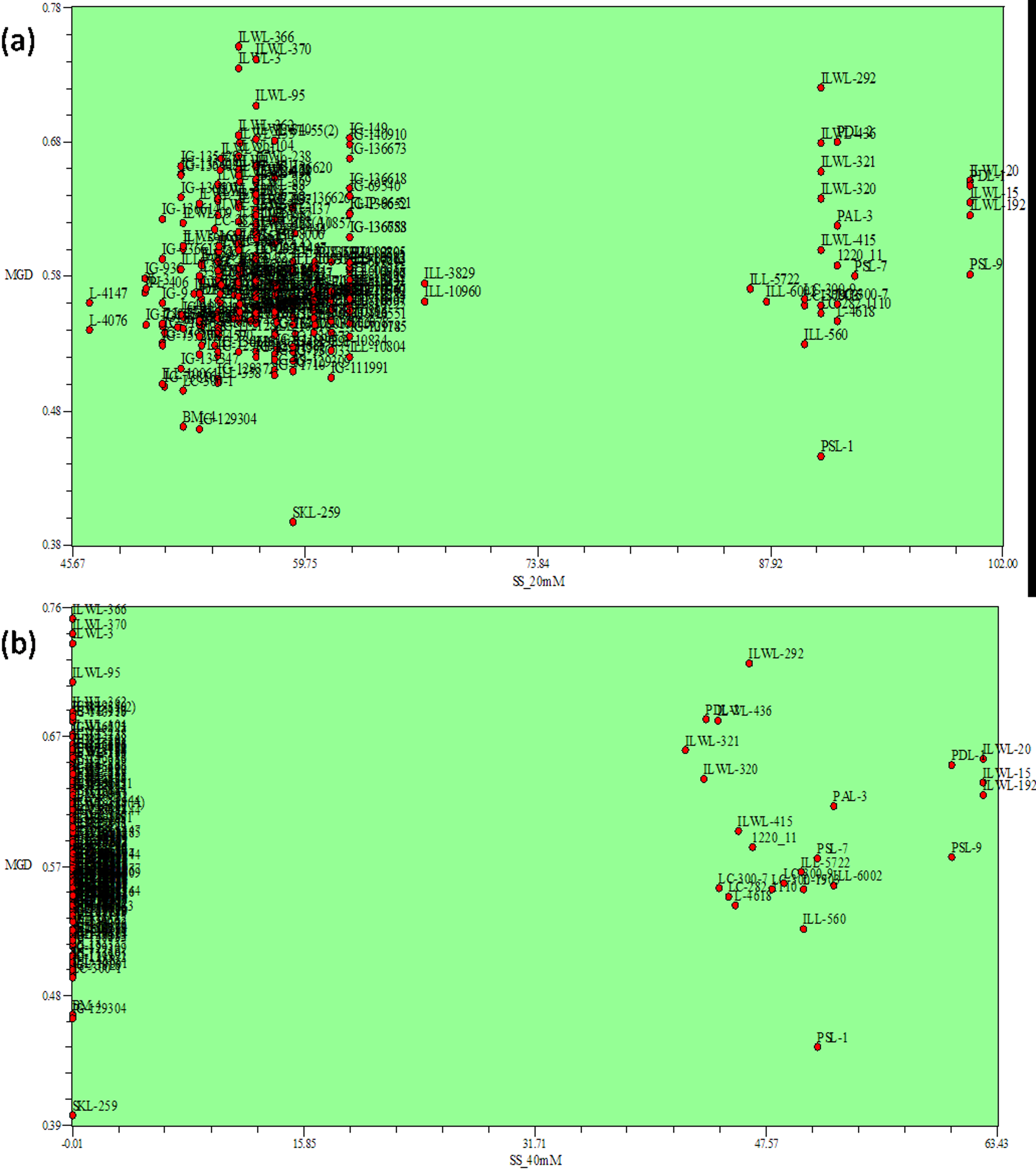

Supplement: S4 Fig — (TIF) [file pone.0199933.s004.tif]
