# Supplementary material for: Evaluation of cultivated and wild genotypes of Lens species under alkalinity stress and their molecular collocation using microsatellite markers
Source: PLoS One. 2018 Aug 13;13(8):e0199933. doi: 10.1371/journal.pone.0199933 (PMC6089424; doi:10.1371/journal.pone.0199933)
Supplement: S2 Table — (DOCX) [file pone.0199933.s006.docx]

**Table S2. Allelic variations and PIC values for 30 SSR markers identified in 285 lentil genotypes.**

| **Marker** | **Major Allele Frequency** | **Allele No** | **Gene Diversity** | **Heterozygosity** | **PIC** |
| --- | --- | --- | --- | --- | --- |
| PLC-35 | 0.572 | 5.000 | 0.590 | 0.109 | 0.535 |
| PLC-05 | 0.388 | 5.000 | 0.700 | 0.133 | 0.648 |
| PLC-30 | 0.451 | 4.000 | 0.626 | 0.018 | 0.550 |
| PLC-39 | 0.774 | 4.000 | 0.375 | 0.004 | 0.342 |
| PLC-81 | 0.607 | 4.000 | 0.535 | 0.042 | 0.463 |
| PLC-91 | 0.577 | 4.000 | 0.560 | 0.074 | 0.488 |
| PLC-100 | 0.298 | 7.000 | 0.771 | 0.091 | 0.733 |
| PLC-104 | 0.656 | 6.000 | 0.533 | 0.018 | 0.500 |
| LC-01 | 0.530 | 7.000 | 0.629 | 0.270 | 0.575 |
| LC-02 | 0.260 | 10.000 | 0.814 | 0.305 | 0.789 |
| LC-16 | 0.493 | 5.000 | 0.567 | 0.081 | 0.473 |
| PBA-LC-221 | 0.598 | 6.000 | 0.589 | 0.067 | 0.550 |
| PBA-LC-222 | 0.440 | 4.000 | 0.665 | 0.039 | 0.604 |
| PBA-LC-368 | 0.418 | 5.000 | 0.674 | 0.035 | 0.613 |
| PBA-LC-377 | 0.530 | 5.000 | 0.631 | 0.014 | 0.577 |
| PBA-LC-379 | 0.456 | 5.000 | 0.600 | 0.095 | 0.518 |
| PLC-51 | 0.702 | 4.000 | 0.465 | 0.000 | 0.423 |
| PBA-LC-376 | 0.744 | 3.000 | 0.400 | 0.000 | 0.349 |
| PBA-LC-117 | 0.754 | 5.000 | 0.405 | 0.039 | 0.375 |
| PBA-LC-118 | 0.470 | 6.000 | 0.649 | 0.014 | 0.583 |
| PBA-LC-404 | 0.498 | 3.000 | 0.585 | 0.000 | 0.499 |
| PBA-LC-1241 | 0.442 | 3.000 | 0.636 | 0.000 | 0.560 |
| PBA-LC-652 | 0.509 | 4.000 | 0.617 | 0.063 | 0.549 |
| PBA-LC-1247 | 0.625 | 4.000 | 0.552 | 0.000 | 0.505 |
| PBA-LC-1401 | 0.460 | 8.000 | 0.700 | 0.246 | 0.659 |
| PBA-LC-1698 | 0.540 | 4.000 | 0.612 | 0.025 | 0.551 |
| PBA-LC-216 | 0.470 | 4.000 | 0.643 | 0.004 | 0.577 |
| PBA-LC-1363 | 0.347 | 5.000 | 0.722 | 0.049 | 0.674 |
| PBA-LC-1308 | 0.740 | 3.000 | 0.410 | 0.000 | 0.364 |
| PBA-LC-383 | 0.554 | 4.000 | 0.601 | 0.039 | 0.542 |
| Mean | 0.530 | 4.867 | 0.595 | 0.062 | 0.539 |

**H, Heterozygosity; PIC, Polymorphism information content.**
