# Supplementary material for: Evaluation of cultivated and wild genotypes of Lens species under alkalinity stress and their molecular collocation using microsatellite markers
Source: PLoS One. 2018 Aug 13;13(8):e0199933. doi: 10.1371/journal.pone.0199933 (PMC6089424; doi:10.1371/journal.pone.0199933)
Supplement: S3 Table — (DOCX) [file pone.0199933.s007.docx]

**S 3.Table . Allelic variations and PIC values for 68 SSR markers identified in 285 lentil genotypes**

| S.no | **Marker** | **Major.Allele.Frquency** | **AlleleNo** | **GeneDiversity** | **Heterozygosity** | **PIC** |
| --- | --- | --- | --- | --- | --- | --- |
| 1 | **PLC-35** | 0.7874 | 4 | 0.3648 | 0.1498 | 0.3458 |
| 2 | **PLC-05** | 0.6005 | 4 | 0.5813 | 0.2065 | 0.5376 |
| 3 | **PLC-30** | 0.7260 | 3 | 0.4038 | 0.0282 | 0.3309 |
| 4 | **PLC-39** | 0.9000 | 3 | 0.1804 | 0.0041 | 0.1648 |
| 5 | **PLC-81** | 0.7723 | 3 | 0.3740 | 0.1071 | 0.3385 |
| 6 | **PLC-91** | 0.6004 | 3 | 0.5257 | 0.0766 | 0.4425 |
| 7 | **PLC-100** | 0.3917 | 6 | 0.7027 | 0.1198 | 0.6478 |
| 8 | **PLC-104** | 0.4388 | 5 | 0.6885 | 0.0510 | 0.6365 |
| 9 | **LC-01** | 0.5037 | 6 | 0.5936 | 0.5746 | 0.5139 |
| 10 | **LC-02** | 0.3045 | 9 | 0.7746 | 0.3580 | 0.7394 |
| 11 | **LC-16** | 0.8673 | 4 | 0.2352 | 0.1420 | 0.2162 |
| 12 | **PBA-LC-221** | 0.6903 | 5 | 0.4771 | 0.0769 | 0.4318 |
| 13 | **PBA-LC-222** | 0.6676 | 3 | 0.4965 | 0.0585 | 0.4436 |
| 14 | **PBA-LC-368** | 0.5783 | 4 | 0.5542 | 0.0602 | 0.4785 |
| 15 | **PBA-LC-377** | 0.5698 | 4 | 0.5784 | 0.0151 | 0.5119 |
| 16 | **PBA-LC-379** | 0.7935 | 4 | 0.3504 | 0.1742 | 0.3251 |
| 17 | **PLC-51** | 0.5529 | 3 | 0.5235 | 0.0000 | 0.4186 |
| 18 | **PBA-LC-376** | 0.8219 | 2 | 0.2927 | 0.0000 | 0.2499 |
| 19 | **PBA-LC-117** | 0.5929 | 4 | 0.5654 | 0.1571 | 0.5056 |
| 20 | **PBA-LC-118** | 0.6233 | 5 | 0.4895 | 0.0186 | 0.3946 |
| 21 | **PBA-LC-404** | 0.7832 | 2 | 0.3396 | 0.0000 | 0.2819 |
| 22 | **PBA-LC-1241** | 0.6415 | 2 | 0.4600 | 0.0000 | 0.3542 |
| 23 | **PBA-LC-652** | 0.6786 | 3 | 0.4848 | 0.1286 | 0.4332 |
| 24 | **PBA-LC-1247** | 0.7511 | 3 | 0.3927 | 0.0000 | 0.3439 |
| 25 | **PBA-LC-1401** | 0.4383 | 7 | 0.6970 | 0.4545 | 0.6482 |
| 26 | **PBA-LC-1698** | 0.4847 | 3 | 0.5457 | 0.0534 | 0.4416 |
| 27 | **PBA-LC-216** | 0.6225 | 3 | 0.5159 | 0.0066 | 0.4400 |
| 28 | **PBA-LC-1363** | 0.5269 | 4 | 0.6305 | 0.0753 | 0.5758 |
| 29 | **PBA-LC-1308** | 0.7432 | 2 | 0.3817 | 0.0000 | 0.3088 |
| 30 | **PBA-LC-383** | 0.5787 | 3 | 0.5394 | 0.0866 | 0.4539 |
| 31 | **PLC-60** | 0.7543 | 2 | 0.3707 | 0.0000 | 0.3020 |
| 32 | **PLC-88** | 0.6179 | 2 | 0.4722 | 0.0500 | 0.3607 |
| 33 | **PBA-LC-373** | 0.4892 | 3 | 0.5432 | 0.0576 | 0.4381 |
| 34 | **PBA-LC-949** | 0.9209 | 3 | 0.1476 | 0.0000 | 0.1401 |
| 35 | **PBA-LC-1351** | 0.6319 | 4 | 0.5139 | 0.1868 | 0.4437 |
| 36 | **PBA-LC-1375** | 0.6484 | 3 | 0.4664 | 0.0313 | 0.3713 |
| 37 | **PBA-LC-278** | 0.9038 | 2 | 0.1738 | 0.1923 | 0.1587 |
| 38 | **PBA-LC-327** | 0.8980 | 2 | 0.1833 | 0.0000 | 0.1665 |
| 39 | **PBA-LC-418** | 0.8488 | 4 | 0.2705 | 0.0349 | 0.2575 |
| 40 | **PBA-LC-333** | 0.2962 | 8 | 0.7604 | 0.2906 | 0.7205 |
| 41 | **PLC-46** | 0.6569 | 7 | 0.5330 | 0.2588 | 0.5016 |
| 42 | **LC-03** | 0.3813 | 11 | 0.7254 | 0.3014 | 0.6846 |
| 43 | **LC-04** | 0.3242 | 12 | 0.8109 | 0.0805 | 0.7883 |
| 44 | **PBA-LC-1480** | 0.4093 | 6 | 0.7078 | 0.1259 | 0.6596 |
| 45 | **PBA-LC-1752** | 0.4071 | 7 | 0.7160 | 0.0474 | 0.6689 |
| 46 | **PBA-LC-1507** | 0.3004 | 7 | 0.7999 | 0.2357 | 0.7721 |
| 47 | **PBA-LC-1751** | 0.4924 | 6 | 0.5923 | 0.0038 | 0.5099 |
| 48 | **PBA-LC-1387** | 0.4955 | 4 | 0.5300 | 0.0000 | 0.4196 |
| 49 | **PBA-LC-260** | 0.8083 | 7 | 0.3362 | 0.0639 | 0.3223 |
| 50 | **PBA-LC-1400** | 0.4271 | 6 | 0.6871 | 0.9397 | 0.6345 |
| 51 | **PBA-LC-1563** | 0.3295 | 6 | 0.7571 | 0.0039 | 0.7172 |
| 52 | **PBA-LC-1316** | 0.2911 | 6 | 0.7680 | 0.0000 | 0.7302 |
| 53 | **PBA-LC-1554** | 0.4748 | 7 | 0.7160 | 0.1473 | 0.6873 |
| 54 | **PBA-LC-1746** | 0.2569 | 9 | 0.8165 | 0.1804 | 0.7917 |
| 55 | **PBA-LC-1478** | 0.3560 | 5 | 0.7335 | 0.0156 | 0.6871 |
| 56 | **PLC-42** | 0.3679 | 6 | 0.7502 | 0.0000 | 0.7114 |
| 57 | **PBA-LC-1689** | 0.8040 | 5 | 0.3423 | 0.0480 | 0.3276 |
| 58 | **PBA-LC-829** | 0.7386 | 3 | 0.4057 | 0.0000 | 0.3524 |
| 59 | **PBA-LC-1403** | 0.5808 | 4 | 0.5627 | 0.0731 | 0.4941 |
| 60 | **PBA-LC-1277** | 0.4295 | 6 | 0.6489 | 0.0726 | 0.5826 |
| 61 | **PBA-LC-900** | 0.4903 | 5 | 0.6012 | 0.1660 | 0.5230 |
| 62 | **PBA-LC-1526** | 0.3275 | 8 | 0.7684 | 0.1589 | 0.7318 |
| 63 | **PBA-LC-1366** | 0.3097 | 7 | 0.7866 | 0.0929 | 0.7554 |
| 64 | **PBA-LC-1525** | 0.4167 | 5 | 0.7131 | 0.0189 | 0.6670 |
| 65 | **PBA-LC-1684** | 0.2811 | 8 | 0.7608 | 0.0453 | 0.7193 |
| 66 | **PBA-LC-1288** | 0.2225 | 9 | 0.8313 | 0.2203 | 0.8090 |
| 67 | **PLC-105** | 0.3879 | 6 | 0.6979 | 0.0514 | 0.6446 |
| 68 | **PBA-LC-1530** | 0.5782 | 5 | 0.5966 | 0.0191 | 0.5488 |
|  | **Mean** | 0.5645 | 4.88 | 0.5491 | 0.1088 | 0.4964 |
