# Supplementary material for: Evaluation of cultivated and wild genotypes of Lens species under alkalinity stress and their molecular collocation using microsatellite markers
Source: PLoS One. 2018 Aug 13;13(8):e0199933. doi: 10.1371/journal.pone.0199933 (PMC6089424; doi:10.1371/journal.pone.0199933)
Supplement: S4 Table — (DOCX) [file pone.0199933.s008.docx]

|  | GM | RL | SL | FWR | FWS | DWR | DWS | survival | Score | Mean genetic distance |
| --- | --- | --- | --- | --- | --- | --- | --- | --- | --- | --- |
| Cluster 1 | 52.851 ^b^ | 52.905 ^c^ | 44.046 ^c^ | 53.75 ^c^ | 47.119 ^b^ | 53.15 ^c^ | 34.645 ^c^ | 1.731 ^c^ | 4.9038^a^ | 0.566 |
| cluster 2 | 51.157 ^b^ | 53.624 ^c^ | 44.269 ^bc^ | 52.579 ^c^ | 47.01 ^b^ | 53.245 ^c^ | 35.262 ^c^ | 1.939 ^c^ | 4.9033^a^ | 0.543 |
| cluster 3 | 52.131 ^b^ | 54.302 ^cb^ | 44.936 ^bc^ | 53.569 ^c^ | 47.381 ^b^ | 53.361 ^bc^ | 34.829 ^c^ | 2.328 ^c^ | 4.878^a^ | 0.555 |
| cluster 4 | 59.63 ^a^ | 60.974 ^a^ | 50.964 ^a^ | 59.916 ^a^ | 52.988 ^a^ | 58.925 ^a^ | 41.784 ^a^ | 18.352 ^a^ | 4.0788 ^c^ | 0.591 |
| cluster 5 | 57.332 ^a^ | 55.137 ^bc^ | 46.506 ^bc^ | 56.669 ^b^ | 46.42 ^b^ | 54.708 ^bc^ | 37.009 ^bc^ | 5.148 ^bc^ | 4.7238^ab^ | 0.593 |
| Cluster 6 | 58.248 ^a^ | 56.368 ^b^ | 46.927 ^b^ | 57.352 ^b^ | 47.531 ^b^ | 55.701 ^b^ | 38.139 ^b^ | 7.109 ^b^ | 4.6293 ^b^ | 0.64 |

**Table S4 : Cluster mean for reduction in germination, root and shoot length, fresh and dry weight of roots and shoots, seedling survival% and mean genetic distance (MGD) under 40mM NaHCO_3_ using total of 68 SSR markers.**

GM-germination; RL-root length; SL-shoot length; FWR-fresh weight root; FWS-fresh weight shoot; DWR-dry weight root; DWS-dry weight shoot; MGD-mean genetic distance

*Values within each column that do not share common letter are significantly different by Duncan’s post- hoc test at P≤0.05*.
